# The *Anopheles* leucine-rich repeat protein APL1C is a pathogen binding factor recognizing *Plasmodium* ookinetes and sporozoites

**DOI:** 10.1371/journal.ppat.1012008

**Published:** 2024-02-14

**Authors:** Natalia Marta Zmarlak, Catherine Lavazec, Emma Brito-Fravallo, Corinne Genève, Eduardo Aliprandini, Manuela Camille Aguirre-Botero, Kenneth D. Vernick, Christian Mitri

**Affiliations:** 1 Institut Pasteur, Université de Paris, CNRS UMR2000, Unit of Genetics and Genomics of Insect Vectors, Department of Parasites and Insect Vectors, Paris, France; 2 Graduate School of Life Sciences ED515, Sorbonne Universities, UPMC Paris VI, Paris, France; 3 Inserm U1016, CNRS UMR8104, Université de Paris, Institut Cochin, Paris, France; 4 Institut Pasteur, Université de Paris, Unit of Malaria Infection & Immunity, Department of Parasites and Insect Vectors, Paris, France; Imperial College London, UNITED KINGDOM

## Abstract

Leucine-rich repeat (LRR) proteins are commonly involved in innate immunity of animals and plants, including for pattern recognition of pathogen-derived elicitors. The *Anopheles* secreted LRR proteins APL1C and LRIM1 are required for malaria ookinete killing in conjunction with the complement-like TEP1 protein. However, the mechanism of parasite immune recognition by the mosquito remains unclear, although it is known that TEP1 lacks inherent binding specificity. Here, we find that APL1C and LRIM1 bind specifically to *Plasmodium berghei* ookinetes, even after depletion of TEP1 transcript and protein, consistent with a role for the LRR proteins in pathogen recognition. Moreover, APL1C does not bind to ookinetes of the human malaria parasite *Plasmodium falciparum*, and is not required for killing of this parasite, which correlates LRR binding specificity and immune protection. Most of the live *P*. *berghei* ookinetes that migrated into the extracellular space exposed to mosquito hemolymph, and almost all dead ookinetes, are bound by APL1C, thus associating LRR protein binding with parasite killing. We also find that APL1C binds to the surface of *P*. *berghei* sporozoites released from oocysts into the mosquito hemocoel and forms a potent barrier limiting salivary gland invasion and mosquito infectivity. Pathogen binding by APL1C provides the first functional explanation for the long-known requirement of APL1C for *P*. *berghei* ookinete killing in the mosquito midgut. We propose that secreted mosquito LRR proteins are required for pathogen discrimination and orientation of immune effector activity, potentially as functional counterparts of the immunoglobulin-based receptors used by vertebrates for antigen recognition.

## Author summary

The parasite that causes malaria must first successfully infect the *Anopheles* mosquito vector to eventually infect a new human host, a process that takes about two weeks. Natural mosquito immunity can kill the parasite and prevent malaria transmission, and understanding this immunity could aid in the development of new malaria control tools. Killing of malaria parasites requires a mosquito blood protein called APL1C, but its mechanism of action remains unclear. We now show that APL1C binds to the rodent malaria parasite, for which it is required for parasite killing, but does not bind to mosquito tissues, nor to the human malaria parasite, which does not require APL1C as a factor for parasite killing. Thus, the function of APL1C may be similar to vertebrate antibodies for pathogen recognition to orient the immune attack.

## Introduction

The innate immune system is an ancient and foundational defense in eukaryotes that often involves proteins with leucine-rich repeat (LRR) domains. Immune LRR protein classes include the well-studied transmembrane proteins such as Toll and Toll-like receptors (TLRs), the intracellular factors such as metazoan and plant nucleotide-binding leucine-rich repeat receptors (NLRs), and also the less studied class of soluble secreted LRR proteins found in invertebrates and jawless agnathan vertebrates [1–5]. The common structural property of LRR proteins is the variation in peptide sequence and LRR repeat number that generates a versatile and highly evolvable binding surface for specific protein to ligand interactions [6–8].

Transmembrane and intracellular TLR and NLR proteins transduce cellular signals upon direct or indirect sensing of pathogen-derived molecules such as virulence factors, flagellin, viral RNA, bacterial DNA, and many others, although the mechanisms of their signal activation by elicitor binding are still not well understood [9–11]. In contrast, the large class of secreted soluble LRR proteins, ubiquitous in invertebrates including mosquitoes, lack evident signaling domains, and their mode of action is not clear [12–15]. It has been speculated that secreted circulating LRR proteins could bind directly to pathogens as discriminant immune receptors [12,16–18]. However, except for the agnathan vertebrate, the lamprey [19], the binding of secreted LRR proteins to pathogens has not yet been demonstrated.

The major African malaria vectors, *Anopheles gambiae* and *Anopheles coluzzii*, have become models for the study of host-pathogen interactions and molecular innate immunity. Female *Anopheles* mosquitoes ingest infected blood carrying Plasmodium gametocytes, which transform into motile ookinetes, traverse midgut epithelial cells, and exit from the basolateral epithelial cell membrane into the extracellular space bounded by the midgut basal lamina. This extracellular space contains mosquito blood serum, the hemolymph, but not blood cells because the porous basal lamina is not permeable to the circulating immune cells, the hemocytes. Once extracellular, the ookinetes form oocysts that grow on the external midgut wall and then rupture to release thousands of sporozoites, which migrate through the hemocoel to invade the salivary glands and render the mosquito infective during subsequent bloodmeals. There are major bottlenecks to parasite development in the mosquito host, because fewer than 1% of ookinetes successfully form an oocyst [20], and fewer than 20% of sporozoites released in the hemocoel invade the salivary glands [21].

A ternary immune protein complex comprised of two LRR proteins, APL1C and LRIM1, and a complement-like thioester protein, TEP1, is an essential component of *Anopheles* immune protection against infection with the model rodent malaria parasite, *Plasmodium berghei* [22–25]. These proteins are secreted by hemocytes as soluble components of the hemolymph [22,23,25–27]. Each of these proteins are members of paralogous gene families [12,16,28]. The APL1 family is comprised of the paralogs APL1A, APL1B and APL1C, sharing ≥ 50% amino acid identity [29].

Initial mosquito immune recognition of malaria parasites must be the prelude to launching a subsequent effector attack for pathogen killing, but the mechanism of parasite recognition and discrimination from mosquito host tissues is essentially unknown. Killing of *P*. *berghei* ookinetes requires the presence of all three proteins, and to date the individual contributions of each protein to the steps of either immune recognition or immune killing are not understood. The TEP1 subunit of the ternary complex lacks specific pathogen binding activity, because TEP1 binds to ookinetes in the presence of the APL1C and LRIM1 partners, but after depletion of APL1C and LRIM1, TEP1 loses the ability to bind to ookinetes and instead binds nonspecifically to mosquito self surfaces within the body cavity [23,24]. These observations point to the LRR subunits as the potential pathogen recognition module orienting the anti-Plasmodium response. However, binding of the LRR proteins to the parasite has not yet been shown, and the role of the LRR subunits in the ternary immune complex has not been previously interrogated.

In addition to the APL1 paralogs and LRIM1, three other LRR proteins have also been shown to protect against Plasmodium midgut infection, namely LRRD7 [30] (AGAP005693, synonyms APL2 [31] and LRIM17 [12]), AGAP007059 and LRIM3 (AGAP007037) [16], as well as two TEP proteins other than TEP1, namely TEP3 and TEP4 [16,17]. Moreover, at least some of the protective LRR and TEP proteins can biochemically interact in different permutations, which implies that a system of combinatorial protein complexes of these and other unknown LRR and TEP factors could potentially generate a high diversity of immune specificities [12,16,17]. However, the functional significance of different LRR and TEP subunit combinations for immune phenotypes has not yet been systematically evaluated.

Here, we employ a basic and general definition of binding specificity, meaning a preferential interaction of APL1C with one target (e.g., an ookinete) and not with the surrounding environment (e.g., mosquito self-tissues). The empirical demonstration of specificity as a preferential interaction does not require knowledge of the precise molecular ligand recognized, which to our knowledge has not previously been shown for any mosquito immune factor acting on malaria parasites.

We report that the LRR proteins APL1C and LRIM1 bind specifically to *P*. *berghei* ookinetes, even when TEP1 expression is silenced, and the protein depleted. APL1C also binds to *P*. *berghei* sporozoites in the hemocoel, and inhibits parasite invasion into the salivary glands, thus directly decreasing parasite transmissibility. In contrast, APL1C does not bind to the human malaria parasite, *P*. *falciparum*, which is consistent with the absence of APL1C protective phenotype against this parasite. The results suggest that mosquito secreted LRR proteins function in pathogen recognition to correctly orient immune effector activity.

## Results

### APL1C protein in hemolymph is induced by bloodmeal and its abundance is not altered by parasite infection

APL1C is a soluble LRR protein secreted into the hemolymph by hemocytes and is not expressed in the midgut [22,23,26,27,32]. Circulating APL1C was measured on by western blot of total hemolymph perfused from pools of ten mosquitoes using anti-APL1C antibody. APL1C levels were significantly higher in hemolymph perfused from mosquitoes 24 h after a non-infective bloodmeal (NBM) as compared to unfed (UF) controls (average 3.27-fold higher APL1C in NBM than UF, range 2.03–5.07-fold increase, p-value = 0.03; S1A and S1B Fig).

**Fig 1:**
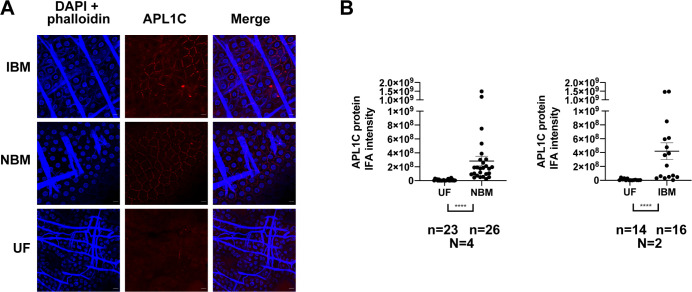
Uninfected bloodmeal induces elevated APL1C protein levels in the hemocoel. **A.** Immunostaining analysis of *P*. *berghei* in mosquito midguts following an infectious bloodmeal (IBM), normal non-infective bloodmeal (NBM) and unfed controls (UF). Images are representative of at least two replicates in each group. APL1C signal depicted in red. Nuclei and actin were stained with DAPI and Phalloidin, respectively (blue). Scale bar is 10 μm. **B.** Quantitative analysis of APL1C signal measured by confocal IFA in hemolymph captured within the extracellular space of IBM, NBM and UF midguts. APL1C IFA signal intensity of each individual midgut is indicated as a dot, bars represent mean with ±SEM. Sample sizes (N) show the number of independent replicate experiments, (n) the total number of midguts dissected across replicates. Data were analyzed by measuring APL1C signal intensity (RawIntDen) using ImageJ v1.52p and the intensity from each midgut was compared between the two conditions by Mann-Whitney test. All statistical differences were first tested independently within replicates (individual p-values in S1 Table), and if individual replicates showed a common trend of change, individual p-values were combined using the meta-analytical approach of Fisher (significance level of Fisher-combined **** p-value <0.0001).

The western blot results using perfused hemolymph were supported by confocal IFA on fixed dissected midguts. Fixation crosslinks and captures cell-free hemolymph proteins in the extracellular space between the cell membrane and the porous basal lamina, allowing their measurement in individual mosquitoes [33,34]. Consistent with the pooled western blot result, confocal IFA of individual midguts indicated that APL1C was significantly more abundant in mosquitoes after NBM as compared to UF controls (p<0.0001, Fig 1 and S1 Table). The presence of *P*. *berghei* in an infectious bloodmeal (IBM) did not alter the amount of APL1C in the hemolymph as compared to a non-infected NBM (Fig 1B, p = 0.2, p = 0.6, and S1 Table). This result indicates that a normal bloodmeal alone is sufficient to induce the maximal level of circulating hemolymph APL1C, and there is no evidence that response to parasite infection plays a role in the level of APL1C induction.

Additional technical controls supported the confocal IFA assay on dissected fixed midguts to quantify relative abundance of APL1C (or potentially other soluble hemolymph proteins) in individual mosquitoes. First, we confirmed that APL1C protein induced by NBM was extracellular to the basolateral membrane of midgut cells by reconstructed confocal optical sections (confocal z-stack, S1C Fig). Second, we confirmed that an injected diffusible molecule the same size as APL1C (70 kDa fluorescent dextran polymer) displayed an equivalent localization pattern to APL1C (S1D Fig). These two results are consistent with APL1C presence in the extracellular space as a soluble protein and not bound to self-tissues. Third, we confirmed that the confocal IFA signal was specific for APL1C protein by silencing APL1C gene expression using treatment with double-stranded RNA (dsRNA), which abolished the confocal IFA signal for APL1C (S2 Fig). Finally, we queried the influence of the previously described enteric microbiome proliferation after a bloodmeal [35,36] by antibiotic treatment of mosquitoes, which did not diminish the induction of APL1C by NBM (S3 Fig), indicating that APL1C induction after NBM is not a response to increased bacterial abundance within the midgut. Thus, mosquito blood feeding induces elevated levels of circulating APL1C protein within the hemolymph, and this increase is not sensitive to the presence of malaria parasites or the enteric microbiota.

### Phagocytic hemocytes are required for normal APL1C protein abundance in hemolymph

APL1C protein prior to secretion is localized in vesicle-like structures within perfused hemocytes and the cultured hemocyte-like 4a3A *A*. *coluzzii* cells (S4A and S4B Fig), similar to the appearance of paralog APL1A [26]. The presence of phagocytic hemocytes was shown to be required for both TEP1 expression and killing of ookinetes [37], but the mechanism of the phagocytic hemocyte requirement was unknown, and the relationship between phagocytic hemocytes and APL1C has not been previously examined.

**Fig 2:**
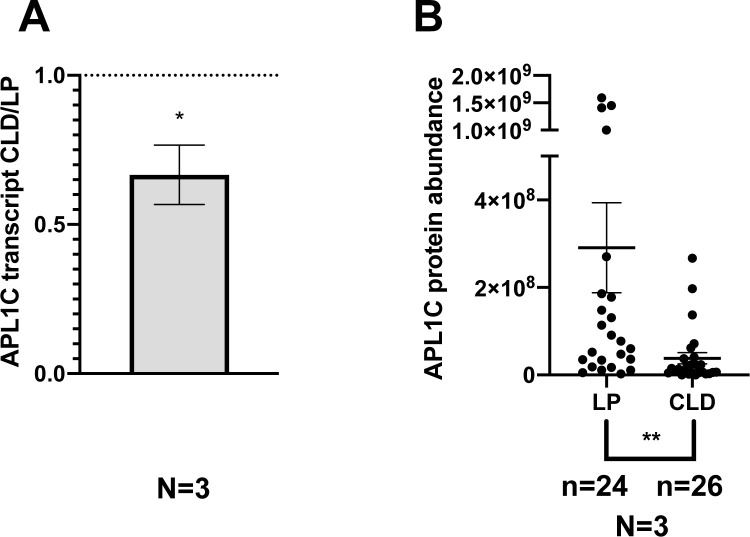
Phagocytic hemocytes are required for normal APL1C protein abundance in hemolymph. **A.** APL1C transcript abundance was reduced in clodronate (CLD) treated mosquitoes as compared to control mosquitoes treated with control empty liposomes (LP, dotted line indicates control). The ratio of normalized APL1C cDNA in CLD versus LP treatments was measured from three biological replicates (N = 3). Graph represents mean with ±SEM of the expression fold change (FC). Significance calculated by unpaired t-test (* p-value<0.05). **B.** APL1C protein in hemolymph requires phagocytic hemocytes. Quantitative analysis of APL1C abundance in hemolymph captured within the midgut extracellular space by confocal IFA of individual dissected midguts after CLD or LP treatment. Analysis details as in Fig 1B. APL1C abundance in each individual midgut is indicated as a point, bars indicate mean ±SEM. Number of independent replicates (N), the total number of midguts dissected across replicates (n). Statistical analysis by Mann-Whitney test using the meta-analytical approach of Fisher (significance level of Fisher-combined ** p-value <0.01).

We chemically depleted phagocytic hemocytes by injecting clodronate liposomes (CLD) or control empty liposomes (LP) prior to a mosquito bloodmeal. The efficiency of phagocyte depletion by CLD treatment was confirmed by the decreased expression of two phagocyte markers, eater and nimrodB2 (S4C Fig). Depletion of phagocytic hemocytes by CLD treatment caused significant reduction of APL1C transcript levels in whole mosquitoes (Fig 2A), and of APL1C protein abundance in the hemolymph (p-value = 0.0087; Fig 2B and S1 Table). Thus, phagocytic hemocytes are an important source of APL1C in the mosquito and are required for normal levels of APL1C gene expression in the whole mosquito and protein abundance in the hemolymph.

### APL1C binds to extracellular *P*. *berghei* ookinetes *in vivo*

It has been hypothesized that the LRR proteins could function as immune recognition receptors [17,38], but binding of the LRR proteins to ookinetes has not been previously demonstrated. Here, we examined *A*. *coluzzii* midguts infected with GFP-expressing *P*. *berghei* for the presence of APL1C protein at 24 h post-infection, the time period when ookinetes traverse and exit from the epithelial cells.

**Fig 3:**
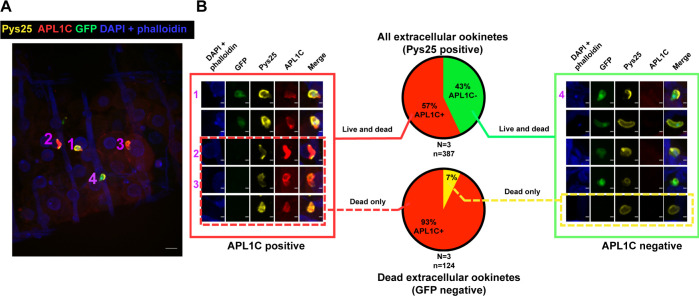
APL1C binds to extracellular *Plasmodium berghei* ookinetes *in vivo*. **A.** Immunostaining of non-permeabilized *P*. *berghei*-infected mosquito midguts 24 h post-infection detects APL1C protein binding to extracellular ookinetes. Extracellular location of parasites is indicated by staining with antibody against Pys25 ookinete surface protein (yellow), APL1C binding is indicated by anti-APL1C antibody staining (red), and live parasites are indicated by expression of GFP (green). Numbered ookinetes indicate representative combinations of attributes: ookinete 1, extracellular (yellow) APL1C-positive (red) and live (green); 2, extracellular, APL1C-postive, and dead (not green); 3, the same as 2; 4, extracellular, APL1C-negative (not red), and live. Nuclei and actin were stained with DAPI and phalloidin, respectively (blue). Scale bar, 10 μm. **B.** The majority of extracellular ookinetes and almost all dead extracellular ookinetes are labelled by APL1C protein. All ookinetes from *P*. *berghei*-infected midguts treated as in panel A were counted and scored for attributes in three biological replicates (N, with 5–14 midguts per replicate), n indicates number of ookinetes scored. Top pie chart depicts the outcome for all extracellular (Pys25-positive) parasites (red pie slice, APL1C-positive extracellular ookinetes, green pie slice, APL1C-negative extracellular parasites), shown as the mean percentage obtained from three replicates. Bottom pie chart depicts the outcome of all dead extracellular (Pys25-positive and GFP negative) parasites (red pie slice, APL1C-positive dead parasites, yellow pie slice, APL1C-negative dead parasites). Parasite images in boxes are unmerged projections of the same numbered parasites shown in panel A. Scale bars of enlarged projection, 2 μm.

Confocal IFA was carried out on non-permeabilized infected midguts using double staining with anti-APL1C antibody and with antibody directed against the ookinete surface protein Pys25 (Fig 3A). Staining with Pys25 is a marker for extracellular ookinetes, to distinguish them from ookinetes in intracellular and midgut lumen locations, while GFP fluorescence distinguishes live ookinetes from the non-fluorescent dead ones regardless of the localization. A majority of extracellular ookinetes (57%) were associated with detectable amounts of APL1C protein (Fig 3B, top pie chart and left panel).

Additional controls confirmed the extracellular location of APL1C-positive ookinetes. First, confocal IFA of permeabilized midguts using anti-APL1C antibody and examined in orthogonal section indicated that the APL1C-labeled parasites were restricted to the basal side of midgut epithelial cells, consistent with an extracellular location (S5 Fig). In contrast, ookinetes that appeared to be intracellular or within the midgut lumen were not labeled by anti-APL1C antibody, indicating that these ookinetes had not yet contacted the APL1C-containing hemolymph. Second, antibody directed against the GFP marker expressed by live parasites labeled 93% of ookinetes in permeabilized midguts (S6 Fig), indicating that permeabilization allows efficient antibody access to parasites located in all regions of the midgut. Thus, the data indicate that APL1C binds to most of the *P*. *berghei* ookinetes that had already exited midgut epithelial cells and contacted the hemolymph.

Dissection and fixation of midguts captures a static snapshot of APL1C labelled ookinetes at a given time point, but the proportion of APL1C labeling could be different at earlier or later times. If LRR binding is required for ookinete killing, then the dead extracellular fraction of ookinetes should represent essentially all ookinetes that were once bound by APL1C and then subsequently killed. Thus, the dead ookinete fraction should summarize the endpoint of APL1C function. Indeed, almost all dead extracellular ookinetes (93%) were positive for APL1C (Fig 3B, bottom pie chart and right panel). A small number of dead extracellular ookinetes were not APL1C labelled (7%), which could represent APL1C label below the detection limit, spontaneous death of unfit parasites, or an infrequent APL1C-independent killing mechanism. Overall, these results indicate that the LRR protein APL1C binds to most of the live extracellular ookinetes at 24 h post-IBM, and almost all dead extracellular ones, consistent with the interpretation that binding of hemolymph APL1C to ookinetes is a necessary event for ookinete killing. Alternatively, these results could also be explained if APL1C simply binds to parasites that are dead or dying for other reasons, although this hypothesis can probably be safely rejected because APL1C is known to be required for ookinete killing as equally as LRIM1 and TEP1. Thus, it seems most likely that APL1C binding to live ookinetes is a required prerequisite for the still unknown mechanism, based on the ternary immune complex, that leads to their killing.

### LRR proteins bind to *P*. *berghei* ookinetes in an *ex vivo* model of hemocoel immunity

**Fig 4:**
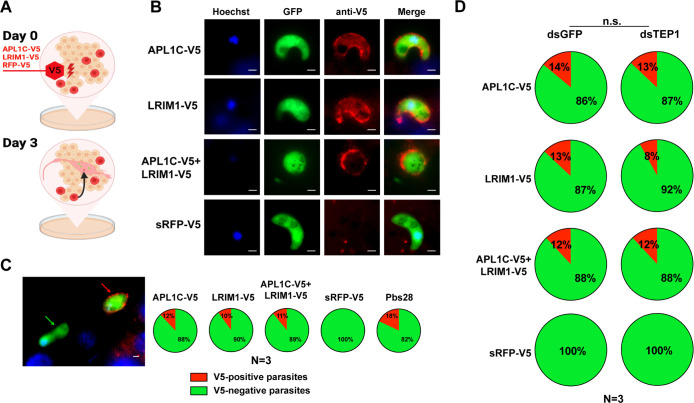
LRR protein binding to *P*. *berghei* ookinetes in an *ex vivo* model. **A.** Summary of the *ex vivo* hemocoel assay. At day 0, the *A*. *coluzzii* hemocyte-like 4a3A cell line was transfected with a plasmid encoding the native APL1C protein including N-terminal signal sequence and with a C-terminal V5 tag (APL1C-V5), a similar construction for LRIM1 including N-terminal signal sequence and C-terminal V5 tag (LRIM1-V5) or control Red Fluorescent Protein gene with the APL1C N-terminal signal sequence and a C-terminal V5 tag (sRFP-V5). At day 3, *P*. *berghei*-infected mosquito midguts were dissected from mosquitoes at 24 h post-infection and incubated for 2 h in culture medium with transfected cells to allow interaction between tagged proteins and GFP-expressing *P*. *berghei* ookinetes. **B.** Immunostaining analysis of infected mosquito midguts treated as depicted in panel A. Staining with anti-V5 monoclonal antibody detects parasites associated with APL1C-V5 and LRIM1-V5 tagged proteins (red). Nuclei were stained with Hoechst 33342 dye (blue). Scale bar, 2 μm. **C.** Percentage of V5-positive parasites (red arrow) and V5-negative parasites (green arrow) after staining with anti-V5 antibodies in each condition were scored and illustrated in pie charts (red pie slice, V5-positive parasites, green pie slice, V5-negative parasites) shown as the mean percentage obtained from three replicates (N, with 3–10 midguts per replicate). Staining with anti-Pbs28 antibodies indicates that 18% of the total GFP parasites were extracellular and exposed to tagged proteins during incubation in culture medium (red, Pbs28-positive parasites, green, Pbs28-negative parasites). Scale bar, 2 μm. **D**. Binding of APL1C and LRIM1 to ookinetes is TEP1-independent. Experimental design as in panel A, but cells were first depleted for TEP1 by treatment with double-stranded RNA (dsTEP1) or treated with a control dsRNA (dsGFP). As in panel C, the proportions of V5-positive and negative parasites after staining with anti-V5 antibodies are indicated in pie charts (red, V5-postive parasites; green, V5-negative parasites), shown as the mean percentage in three replicates (N, with 3–10 midguts per replicate). The percentage of V5-positive and negative parasites from each replicate were compared between dsTEP1 and dsGFP treatments by chi-square test (n.s., not significant). Panel A created with BioRender.com.

To extend the observations of LRR binding to *P*. *berghei* ookinetes, we generated an *ex vivo* model of the *P*. *berghei*-infected mosquito hemocoel (Fig 4A). To establish the model hemocoel, hemocyte-like 4a3A cells were transfected with plasmids expressing native sequences of LRR proteins APL1C and/or LRIM1 including N-terminal signal sequences and bearing a C-terminal V5 epitope tag (APL1C-V5 and LRIM1-V5, respectively). As a negative control, cells were transfected with a construct expressing RFP fused with the APL1C N-terminal signal sequence and tagged with V5 (sRFP-V5). Immunoblotting of culture medium confirmed that sRFP-V5 was secreted from cells whereas the RFP-V5 protein without signal sequence was retained in the cell cytoplasm (S7A Fig). APL1C-V5 and LRIM1-V5 were also secreted into the culture medium and formed high molecular weight complexes under non-reducing conditions, consistent with predicted sizes of APL1C or LRIM1 homodimers and APL1C/LRIM1 heterodimers (arrowheads, S7B Fig), in addition to the monomeric forms expected at 88kDa and 60kDa for APL1C and LRIM1, respectively (arrows, S7B Fig).

Next, to incorporate *P*. *berghei* infection into the *ex vivo* model, midguts were dissected from mosquitoes at 24 h after bloodmeal infection with GFP-expressing *P*. *berghei*, and the infected midguts were directly incubated without fixation in the culture medium of the transfected 4a3A cells for 2 h. The midguts were then fixed, permeabilized, and subjected to IFA with anti-V5 antibody to detect tagged LRR proteins that bound to ookinetes. Both APL1C-V5 and LRIM1-V5 were colocalized with live (GFP-positive) ookinetes (Fig 4B). Similar proportions of GFP-positive parasites were associated with APL1C-V5 and LRIM1-V5, 12% and 10% respectively, whereas no V5-positive signal was observed in midguts incubated with medium from cells expressing sRFP-V5, indicating that the colocalization was specific for the LRR proteins (Fig 4C). In either case, more than 85% of ookinetes were V5-negative, likely because at 24 h post-infection, all the ookinetes have not yet fully migrated through the midgut epithelium to exit into the extracellular space facing the basal lamina, and therefore would not be accessible to antibodies in the culture medium. As a control for ookinete exposure to the medium, non-permeabilized infected midguts were immunostained with an antibody directed against the ookinete surface protein Pb28 [39], which indicated that only 18% of parasites were Pb28-positive. Therefore, the expressed LRR-V5 proteins were associated with a majority (56–67%) of ookinetes exposed to the medium (Fig 4C). This proportion is equivalent to the fraction of APL1C-positive ookinetes (57%) measured in the *in vivo* infection experiments presented above, confirming the fidelity of the *ex vivo* model.

A similar proportion of labeled parasites was detected in midguts incubated in medium from cells co-transfected with both APL1C-V5 and LRIM1-V5, as compared to the single transfections (11%, Fig 4C), which is consistent with the interpretation that LRR heterodimers were bound to ookinetes. This was tested *in vivo*, where IFA co-immunostaining with APL1C and LRIM1 antibodies indicated simultaneous presence of both LRR proteins on the ookinete surface, strongly suggesting their presence as a heterodimer (S8 Fig). Binding as LRR homodimers cannot be excluded, and homodimer formation has been demonstrated biochemically, but previous observations support LRR heterodimers as the predominant functional form [22,23,25–27].

It is known that TEP1 binding to ookinetes requires the presence of APL1C and LRIM1 [22–24]. To query the reciprocal case, whether LRR protein binding to ookinetes requires the presence of TEP1, we depleted TEP1 protein in 4a3A cells by treatment with dsRNA directed against TEP1 before transfection with constructs expressing LRR-V5 proteins. Immunoblotting confirmed that TEP1 gene silencing depleted the protein by 75 to 79% (S9 Fig). Although RNAi-mediated gene silencing does not entirely abolish target gene transcripts, the observed level of depletion indicated efficient silencing of TEP1. Importantly, the depletion of TEP1 was comparable to that previously obtained for depletion of APL1C or LRIM1, in which deposition of TEP1 onto *P*. *berghei* ookinetes was completely eliminated [24]. In contrast, we found that depletion of TEP1 to an equivalent efficiency did not alter the proportion of ookinetes that were bound by LRR-V5 proteins (Fig 4D).

These results suggest that APL1C and LRIM1 can specifically bind to *P*. *berghei* ookinetes without involvement of TEP1. This interpretation is consistent with the previous observation that the APL1C/LRIM1 heteroduplex is biochemically stable in hemolymph after depletion of TEP1 while conversely, after depletion of APL1C and/or LRIM1, TEP1 protein disappears from the hemolymph and binds nonspecifically to mosquito self surfaces in the hemocoel [22–24]. Taken together, the current and previous observations support the interpretation that APL1C and LRIM1 mediate immune recognition of the parasite surface to orient and target eventual parasite destruction, a step that also equally requires presence of TEP1.

### APL1C does not bind to nor kill ookinetes of *P*. *falciparum*

While APL1C activity is essential for killing of *P*. *berghei* ookinetes, it displays no detectable influence on mosquito infection prevalence of the human malaria parasite, *P*. *falciparum* [16,29,38,40]. Instead, the control of *P*. *falciparum* infection requires activity of the APL1 family paralog, APL1A. The mechanism of pathogen specificity among the APL1A and APL1C is not understood. However, the proposed mechanism in which APL1C binding is required for *P*. *berghei* recognition leads to the prediction that APL1C should not display similar binding activity to *P*. *falciparum* parasites as it displays for *P*. *berghei*, because it is not protective against *P*. *falciparum*.

**Fig 5:**
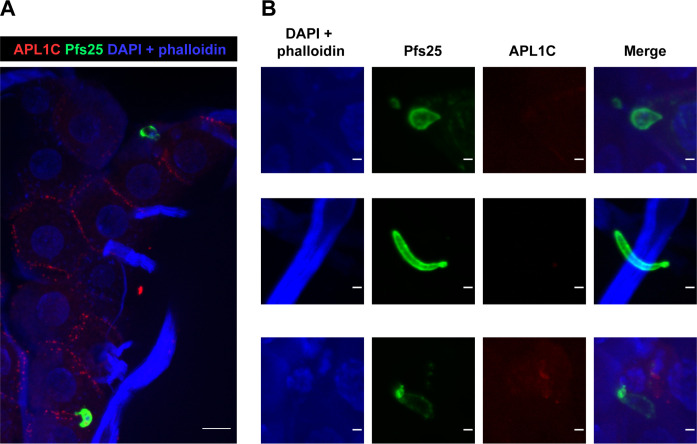
APL1C does not bind to ookinetes of *P*. *falciparum*. Confocal immunostaining analysis of non-permeabilized midguts at 24 h post-infection with *P*. *falciparum*. **A**. The projection depicts two midgut ookinetes stained with Pfs25 protein (green), indicating that they were extracellular, but not associated with APL1C protein (red). As a positive control for APL1C detection, the IFA signal of hemolymph APL1C in the extracellular space can be seen, as in Fig 1. Scale bar, 10 μm. **B**. Enlarged projections depict ookinetes that are stained with Pfs25 but not APL1C, representative of the n = 47 ookinetes observed, none of which were labelled by APL1C. The bottom row depicts a diffuse APL1C signal that was occasionally observed in proximity to *P*. *falciparum* ookinetes. Nuclei and actin were stained with DAPI and phalloidin (blue). Scale bar, 2 μm.

We assayed APL1C binding to *P*. *falciparum* ookinetes under the same conditions used to detect *in vivo* APL1C binding to *P*. *berghei* ookinetes. Midguts were dissected from mosquitoes 24 h after a *P*. *falciparum*-infected bloodmeal, and IFA was performed on non-permeabilized midguts using antibodies directed against APL1C. The *P*. *falciparum* parasite strain was not fluorescent, and ookinetes were detected by staining with antibody against the *P*. *falciparum* ookinete surface protein, Pfs25. Because the midguts were non-permeabilized, Pfs25 antibody labeling only detects ookinetes that were extracellular and exposed to hemolymph within the extracellular space. Of 55 midguts examined, 21 midguts (38%) were infected with a total of 47 extracellular (Pfs25 positive) *P*. *falciparum* ookinetes. Experimental infection with *P*. *falciparum* is less efficient than the model *P*. *berghei*, typically yielding lower mosquito infection prevalence and intensity. Of the 47 extracellular ookinetes observed, none were bound by detectable amounts of APL1C protein (Fig 5). The signal of hemolymph APL1C in the extracellular space (as in Fig 1) is visible in the low magnification image (Fig 5A) and serves as a positive control for function of the APL1C antibody. Interestingly, a diffuse APL1C signal was occasionally observed next to *P*. *falciparum* ookinetes (Fig 5B, bottom row), which suggests the possibility that a secreted product from *P*. *falciparum* ookinetes may be recognized by APL1C with low efficiency. This will require further observations, but if so, the ookinete secretion could be an immune decoy, and/or could also explain a weak effect of APL1C that was reported for *P*. *falciparum* infection intensity but not prevalence under certain infection conditions [40].

Experimental infections with the non-natural *P*. *berghei* model tend to generate higher parasite infection intensity than natural infection levels of *P*. *falciparum*. Therefore, it is possible that the artificially high infection levels of *P*. *berghei* could potentially induce APL1C binding, while the low infection intensity of *P*. *falciparum* would not reach a threshold to induce APL1C binding. To test this possibility, we controlled for a possible influence of high *P*. *berghei* infection intensity on APL1C binding efficiency in two ways. First, we separately analyzed APL1C binding only in the *P*. *berghei* infections with lowest ookinete infection intensity, those with fewer than 10 ookinetes per midgut, which is comparable to *P*. *falciparum* infection intensity levels. APL1C bound to 63% of ookinetes in these low intensity *P*. *berghei* infections (S10A and S10B Fig, 12 low infection intensity midguts carrying n = 36 extracellular Pys25-positive ookinetes, APL1C binding observed to n = 23 (63%) of counted parasites). This is comparable to the 57% APL1C binding rate observed in all examined *P*. *berghei* infected midguts, the majority with higher infection intensities (Fig 3). Thus, APL1C binding efficiency to *P*. *berghei* ookinetes is not an effect or consequence of high parasite numbers.

Second, we specifically tested the functional immune protective effect of APL1C in *P*. *berghei* infections at low infection levels comparable to the *P*. *falciparum* infections by silencing APL1C expression in mosquitoes infected with *P*. *berghei* at low oocyst infection intensity of median 2 oocysts in the dsGFP control. APL1C silencing causes significantly higher infection prevalence as well as intensity as compared to dsGFP controls (prevalence and intensity effects, P-value<0.0001), indicating that APL1C is equally required for protection from *P*. *berghei* infections equivalent to those of *P*. *falciparum*. Thus, infection intensity is not a factor influencing the efficiency of APL1C binding to *P*. *berghei* ookinetes nor to the protective function of APL1C against *P*. *berghei* infection. Overall, these results indicate that, under the same conditions in which APL1C efficiently binds to *P*. *berghei* ookinetes, it does not bind to *P*. *falciparum* ookinetes, and therefore APL1C displays discrimination for specific parasite binding that correlates with its protective phenotype.

### APL1C activity in the hemocoel limits *P*. *berghei* sporozoite invasion of salivary glands

Like the ookinete, the sporozoite stage is also directly exposed to hemolymph, beginning with the rupture of midgut oocysts approximately 12 d post-infection. Invasion of sporozoites into the salivary glands renders the mosquito infectious for malaria transmission. However, analogous to midgut ookinetes, the majority of sporozoites are destroyed during their migration through the hemocoel, and only a small proportion of the thousands released per oocyst successfully invade the salivary glands [21]. The mechanism of sporozoite destruction in the hemocoel remains unknown.

**Fig 6:**
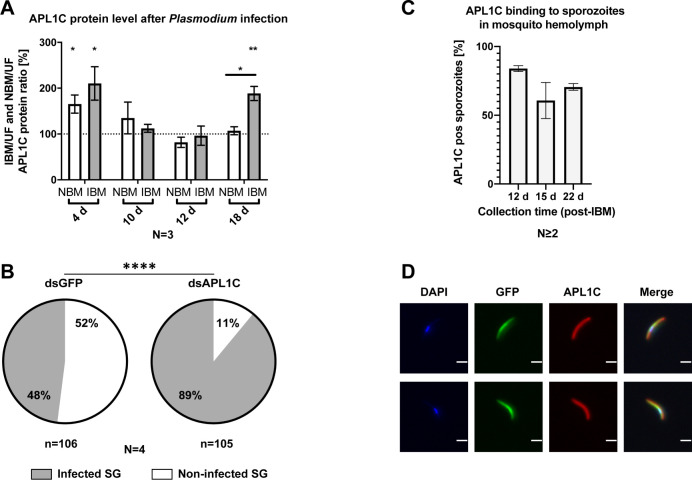
APL1C activity in the hemocoel limits *P*. *berghei* sporozoite invasion of salivary glands. **A.** APL1C protein level increases transiently in mosquitoes after either non-infective bloodmeal (NBM) or *P*. *berghei* infectious bloodmeal (IBM), and in IBM mosquitoes again during sporozoite release. APL1C levels in whole mosquitoes were determined by western blot using anti-APL1C antibody normalized to control anti-GADPH antibody. Time points correspond to presence in IBM mosquitoes of young oocysts (4 d), mature oocysts before sporozoite release (10 d), early sporozoite release (12 d) and late sporozoite release (18 d). Signals were compared to unfed (UF) controls, defined as 100% (dashed line). Graph represents mean with ±SEM of the fold-change between IBM/UF or NBM/UF, from three biological replicates (N = 3). Data were analyzed by unpaired t-test (significance levels: * p-value<0.05; ** p-value <0.01). **B.** APL1C depletion strongly increased the fraction of mosquitoes with infected salivary glands. Sporozoites were counted in the salivary glands dissected from individual mosquitoes after prior treatment with dsAPL1C or dsGFP. Each mosquito was scored as sporozoite-infected (gray) or non-infected (white) in four biological replicates (N = 4). Salivary gland infection prevalence in each replicate was compared between the two dsRNA treatment conditions by chi-square test (individual p-values in S2 Table) and were combined using the meta-analytical approach of Fisher (significance level of chi-square **** p-value <0.0001). **C-D.** APL1C protein binds sporozoites in the mosquito hemocoel**. C.** Sporozoites were perfused from mosquitoes at different times post-infection and analyzed by flow cytometry using anti-APL1C antibody. Graph represents the mean percentage of APL1C-positive sporozoites from at least two biological replicates, bars represent mean with ±SEM. **D.** APL1C binding to sporozoites was confirmed by IFA of sporozoites perfused from mosquitoes at 17 d post-infection labeled with anti-APL1C antibody. GFP-expressing sporozoites (green) were observed coated with APL1C protein (red). Nuclei were stained with DAPI (blue). Scale bar, 5 μm.

To examine whether APL1C also plays a role in host defense against sporozoites, we first determined whether APL1C abundance is altered by the release of sporozoites from rupturing oocysts. APL1C protein abundance was measured by western blot in pools of mosquitoes at four time points during *P*. *berghei* development: young oocysts at 4 d post-bloodmeal, late oocysts before sporozoite release at 10 d post-bloodmeal, early sporozoite release at 12 d post-bloodmeal, and late sporozoite release at 18 d post-bloodmeal (Figs 6A and S11). At 4 d post-bloodmeal, APL1C protein was increased in both IBM and NBM mosquitoes as compared to UF, but there was no difference between IBM and NBM. This result is consistent with measurement of APL1C in individual mosquitoes using confocal IFA (Fig 1 and S1 Table), indicating that the initial induction of APL1C protein was dependent only on the bloodmeal and is not responsive to *P*. *berghei* infection. At 10 d and 12 d post-bloodmeal, APL1C abundance in both IBM and NBM mosquitoes was equivalent to the UF controls, indicating that APL1C levels decrease to baseline after decay of the bloodmeal effect. However, at 18 d post-bloodmeal during sporozoite release, APL1C protein abundance was significantly higher in IBM as compared to either NBM or UF mosquitoes, the latter two of which were not different from each other (Fig 6A).

Thus, unlike the initial bloodmeal infection, in which APL1C levels are sensitive to bloodmeal but not the presence of parasites, at 18 d post-IBM the signaling mechanism that augments APL1C abundance appears directly sensitive to the presence of hemocoel sporozoites. This observation suggested a potential protective role, and to test for APL1C influence on sporozoite viability, mosquitoes were depleted for APL1C protein by treatment with dsAPL1C or control dsGFP at 9 d post-IBM, which is approximately 3 d before the beginning of sporozoite release. APL1C transcript and protein depletion was efficient until at least 10 d after dsRNA treatment (S12 Fig). The pair of salivary glands was dissected from individual mosquitoes and sporozoites per mosquito were counted at 19 d post-IBM. APL1C-depleted mosquitoes displayed strongly increased salivary gland infection prevalence, defined as the proportion of mosquitoes with infected salivary glands (p-value<0.0001, Fig 6B and S2 Table). This result means that, in a significant proportion of infected mosquitoes, the normal activity of APL1C protects the salivary glands from the invasion of any detectable sporozoites. There was no effect of APL1C depletion on salivary gland infection intensity, defined as the numbers of salivary gland sporozoites in mosquitoes with infected glands; S2 Table).

The simplest model to explain APL1C inhibition of sporozoite invasion into the salivary glands is analogous to the one proposed for ookinete immunity, in which LRR proteins recognize and bind the parasite, and in conjunction with TEP1 cause parasite death. Therefore, we first tested whether APL1C can bind to sporozoites. Hemocoel sporozoites were perfused from mosquitoes at three different time points post-IBM (12 d, 15 d and 22 d). APL1C binding to the sporozoites was measured by flow cytometric analysis using anti-APL1C antibody (S13A–S13D Fig). The antibody detected APL1C protein present on the sporozoite that was naturally acquired from their exposure to mosquito hemolymph prior to perfusion. The majority of perfused hemocoel sporozoites were labelled with APL1C at all analyzed time points (84% for 12 d, 61% for 15 d and 71% for 22 d, Figs 6C and S13E). APL1C binding was also confirmed visually by IFA with anti-APL1C antibody on perfused hemocoel sporozoites (Fig 6D). The specificity of APL1C binding was confirmed by silencing APL1C expression in mosquitoes followed by sporozoite perfusion and IFA. Almost no perfused sporozoites from APL1C-depleted mosquitoes were labelled with APL1C antibody (0.95%, n = 210; S14 Fig), while 100-fold more sporozoites (94%) were labeled with APL1C when perfused from dsGFP-treated control mosquitoes.

Next, we examined the mechanism by which APL1C limits salivary gland infection. Two possible models are that i) APL1C binding may play a role in the previously described sporozoite killing prior to salivary gland invasion [21], which would be analogous to ookinete killing in the midgut, or ii) APL1C binding impedes salivary gland invasion by a mechanism that does not directly rely on immediate sporozoite killing. To distinguish between these models, APL1C was depleted by dsRNA treatment of mosquitoes at 9 d post-IBM (prior to sporozoite release from oocysts), and mosquitoes were perfused individually to recover circulating sporozoites at 17 d post-IBM (after sporozoite release). There was no effect of APL1C depletion on the number of circulating hemocoel sporozoites as quantified by flow cytometry (S15A Fig and S1 Table). Efficient APL1C gene silencing at the time of perfusion was confirmed in mosquitoes (S15B Fig). A similar analysis performed for LRIM1 indicated that activity of this LRR also did not influence sporozoite numbers in the mosquito hemocoel (S15C and S15D Fig and S1 Table).

Thus, APL1C activity limits salivary gland infection of sporozoites by a mechanism that does not lead to a detectable physical reduction of the number of circulating sporozoites in the hemolymph, for example by direct sporozoite destruction. It might be expected that the control (dsGFP-treated) mosquitoes should display an augmentation of circulating hemolymph sporozoites as compared to APL1C-depleted mosquitoes because of the sporozoites that were inhibited from invading the salivary glands by APL1C activity and therefore remained circulating in the hemocoel, but there was not a difference in circulating sporozoite numbers (S15A Fig). However, as previously reported, fewer than 20% of all hemocoel sporozoites invade the salivary glands [21]. Here, we observed 48% salivary gland infection prevalence in dsGFP-treated control mosquitoes and 89% in APL1C-depleted mosquitoes, yielding a difference of 41% of the glands that were protected from any detectable invasion due to APL1C activity. Thus, we infer that there would be ~8% more circulating sporozoites in absolute numbers in the dsGFP controls (i.e., 41% of the total 20% invading sporozoites), and conclude that this would likely be too small a difference in absolute numbers to be reliably detected in perfused hemolymph.

Additional evidence also indicates that the anti-sporozoite mechanism of APL1C differs from APL1C-dependent ookinete killing in the mosquito midgut. By analogy to the ookinete-killing system, the involvement of a TEP partner would be expected. Therefore, we queried the role of the two TEP family members, TEP1 and TEP3, that have been shown to promote the killing of *P*. *berghei* ookinetes [16,17,22,23]. It was previously suggested that TEP1 does not bind to sporozoites [41]. Depletion of TEP1 and TEP3 by silencing individually or simultaneously at 9 d post-IBM did not influence sporozoite infection of salivary glands (S16 Fig and S2 Table). Therefore, neither of these TEP family members involved in *P*. *berghei* ookinete killing are required for sporozoite immunity.

These results demonstrate that APL1C protein in the hemolymph is correlated in time with, and presumably induced by, sporozoite release from oocysts, that APL1C protein binds to circulating sporozoites in the hemocoel, and that APL1C activity strongly decreases the proportion of mosquitoes with sporozoite-infected salivary glands. The anti-sporozoite effect of APL1C does not appear to directly cause physical elimination of sporozoites from mosquito hemolymph. Instead, APL1C inhibits their ability to invade the salivary glands, potentially by coating and blocking sporozoite ligands necessary for invasion, and/or inhibiting sporozoite motility necessary for gland invasion. Nevertheless, the inability of sporozoites to invade the salivary glands would likely lead to their eventual elimination from the hemolymph, because they have no other tissue target, which would be a downstream consequence but not direct effect of APL1C activity. Finally, analysis of potential APL1C immune partners revealed that different TEP family members may be required for anti-sporozoite immunity, distinct from those involved in *P*. *berghei* ookinete killing, or alternatively sporozoite destruction could be mediated by a mechanism that does not include a TEP family factor. Further work will be required to distinguish between these possibilities.

## Discussion

Here, we show that an invertebrate secreted LRR protein, APL1C, is a binding factor of *Plasmodium berghei* parasites at two developmental stages, midgut ookinetes and hemocoel sporozoites. It has long been known that APL1C is required, along with LRIM1 and TEP1, for *P*. *berghei* ookinete killing in the mosquito midgut, and these results provide the first functional explanation for the APL1C role. We also find that APL1C activity strongly decreases the proportion of salivary glands infected with sporozoites, thereby directly limiting the proportion of infectious mosquitoes competent to transmit the parasite to the next host. In both parasite stages, APL1C binding is the common feature and could underlie specific pathogen recognition, which is not yet understood in the mosquito response to malaria (or any other) infection.

Unlike many immune LRR proteins in metazoans and plants, which are transmembrane or intracellular proteins with signal transduction domains, APL1C is a soluble, secreted protein with a signal peptide, and lacks an evident signal transduction domain [8,12]. The best characterized soluble LRR immune proteins are the variable lymphocyte receptors (sVLR) of jawless vertebrates [5,42–44]. In the lamprey, three germline VLR genes (VLRA, VLRB, VLRC) generate immunological diversity through a gene conversion-like mechanism giving rise to diversity in cellular and humoral LRR-bearing receptors, analogous to the immunoglobulin (Ig)-based antigen receptors used by jawed vertebrates. Secreted VLRB is an LRR antibody that mounts an antigen-specific immune response, including opsonization and activation of the lamprey complement pathway [19,45]. The VLRs serve as an intriguing comparison for insect LRR proteins mainly because the LRR domain is the specific antigen binding surface of the VLRs, although these vertebrate proteins are functionally more complex than and may not be a good model for insect LRR proteins because the VLRs are literal antibodies within a system of cellular immune memory.

There is no evidence for somatic rearrangement of mosquito or insect LRR genes. However, the APL1 gene family displays structural diversity consistent with a high frequency of paralogous gene conversion and are among the most polymorphic genes in the *Anopheles* genome [8]. Like the sVLRB Ig-like multimers, the APL1 family and other LRR proteins can form homo- and heterocomplexes through cysteine disulfide bonds [16,17,23,46]. Based on the potential number of distinct LRR heterocomplexes that could form and factoring in the high genetic polymorphism of at least the APL1 genes, our results raise the possibility that a potentially large number of combinatorial repertoires of functional immune complexes may generate numerous pathogen binding and recognition specificities. Formation of the APL1C/LRIM1 heteroduplex is required for *P*. *berghei* ookinete killing in conjunction with the presence of TEP1 [22,24]. Although it is thought that the TEP1 partner may be a killing effector due to its molecular similarity to complement, the specific functional contribution of each of the three molecular partners of the immune complex is not understood, in part because inactivation of any single partner dominantly abolishes the only known phenotype of the active immune complex, which is killing of *P*. *berghei* ookinetes.

In particular, the previously described function for the LRR heteroduplex in *P*. *berghei* ookinete killing was that depletion of either LRR protein of the APL1C/LRIM1 heteroduplex causes spontaneous and nonspecific TEP1 deposition on self-tissues within the mosquito body cavity [23,24]. Conversely, depletion of TEP1 does not diminish hemolymph levels of the LRR heteroduplex. The current results demonstrate that APL1C binds specifically to *P*. *berghei* ookinetes and sporozoites but not to *P*. *falciparum* ookinetes. The binding discrimination of APL1C mirrors previous observations that APL1C limits midgut infection by the rodent malaria species *P*. *berghei* and *Plasmodium yoelii* but not the human parasite *P*. *falciparum*, whereas the paralog APL1A is required for protection from *P*. *falciparum* but not the rodent parasites [16,29,38]. The current results are consistent with a model in which the APL1C/LRIM1 heteroduplex is required for the recognition of *P*. *berghei* ookinetes and at least APL1C for *P*. *berghei* sporozoites, and in which the selective protective phenotype of APL1C for *P*. *berghei* is based on its distinct recognition capacities. Parasite binding of APL1A has not been tested, but it was shown to also form a disulfide-bonded heterodimer with LRIM1, leading to the prediction that APL1A might bind to *P*. *falciparum*, against which it protects, but not the rodent parasite species, for which it does not display a phenotype [16].

The current results are compatible with the direct and autonomous binding of APL1C in the LRR heteroduplex to ookinetes, but do not rule out alternative explanations. Specifically, it cannot be excluded that other known or unknown protein partners may bind to ookinetes before or in partnership with the APL1C/LRIM1 heteroduplex. The sum of previous observations indicates that the two LRRs and TEP1 are all equally required for ookinete killing, but our data suggest that TEP1 presence is not necessary for LRR binding, because LRR binding still occurs after dsRNA-mediated silencing of TEP1 transcript and protein. It could be argued that residual TEP1 produced after silencing is sufficient to promote LRR binding, or that other TEP family paralogs could potentially complement the absence of TEP1. Nevertheless, the level of TEP1 depletion we obtained was equivalent to that seen in previous studies in which depletion of TEP1 did not alter hemolymph abundance of the LRRs, while depletion of either APL1C or LRIM1 abolished TEP1 presence in the hemolymph as well as its binding to ookinetes [22–24]. Thus, our results show that the LRRs still display specific ookinete binding activity after depletion of TEP1, whereas the reverse was not true because TEP1 is not present on ookinetes or in the hemolymph after equivalently efficient depletion of the LRRs. Taking published and current results together, the only property that distinguishes the three proteins appears to be that APL1C and LRIM1 display stability in the hemolymph and ookinete binding after depletion of TEP1, while TEP1 is unstable in the hemolymph and does not bind ookinetes if either of the two LRRs are depleted. The simplest interpretation is that the deposition of APL1C/LRIM1 heteroduplex mediates specific *P*. *berghei* pathogen recognition even without TEP1, but that the presence of all three proteins is required to effectuate *P*. *berghei* ookinete killing.

Further dissection of independent activity of the three proteins by controlling for or eliminating the others will be challenging, in part because the presence of all three partners is essential for *P*. *berghei* ookinete killing. Here we enumerate some possibilities and their limitations. To control for the TEP1 phenotypic contribution, a TEP1 loss-of-function mutant could be generated by CRISPR/Cas9-mediated deletion of the TEP1 gene in mosquitoes, but this would still leave 14 other TEP paralogs that could potentially complement loss of TEP1. Deletion of all 15 TEP genes by CRISPR/Cas9 would be impractical. To conclusively confirm autonomous APL1C/LRIM1 binding to ookinetes, a non-mosquito or even non-insect cell line could be transfected with plasmids expressing the LRR genes followed by incubation of the cells with infected midguts as in the above *ex vivo* system, but this would not eliminate the possibility that the mosquito LRRs could interact with unknown but functionally analogous proteins from the host cells. In addition, LRR protein post-translational modifications would be incorrect in non-insect cells, and finally, previous observations suggest that products secreted into the culture medium by non-insect cells would be reasonably likely to kill malaria ookinetes and/or mosquito midgut cells during co-culture [47,48]. Lastly, it could be possible to express the two LRR proteins from mosquito cells, purify them to homogeneity, and incubate with infected midguts in a cell-free *ex vivo* system, but this would not exclude the possibility that unknown proteins had already bound to ookinetes in the infected mosquitoes from which the midguts were dissected. Instead of infected midguts, generation of *P*. *berghei* ookinetes in vitro was described [49], but the ookinetes are not viable and die rapidly. Thus, the inextricable joint function of the three proteins for ookinete killing appears to present a major barrier to dissecting their independent contributions, with requirement for increasingly complex experiments and likely diminishing returns of further insight regarding their individual functional roles.

The mosquito immune response to circulating malaria sporozoites in the hemocoel has been little studied. The hemolymph constitutes a hostile environment for sporozoites once they are released from oocysts, leading to their rapid destruction by a still uncharacterized mechanism [21]. Here, we identify APL1C as an essential component required for the limitation of *P*. *berghei* sporozoite invasion of salivary glands. However, hemocoel sporozoite immunity is mechanistically distinct from midgut ookinete killing, because neither of the known TEP partner molecules involved in ookinete killing are required, and APL1C activity does not lead directly to immediate sporozoite killing. It is not clear why ookinetes and sporozoites, both exposed to the hemolymph compartment, would be recognized by the same soluble LRR protein, APL1C, but require interaction with different protein partners for the immune phenotype. The answer could be related to potential differences in cell surface characteristics, which might require distinct recognition strategies.

Hematophagy, an arthropod adaptation that is more than 40 million years old [50], also caused arthropod exposure to novel microbial profiles. In addition to tolerating the large increase in abundance of the enteric bacterial microbiome after a bloodmeal, arthropods also required defenses against a new repertoire of blood-borne microbes, some of which in turn adapted to the arthropod to gain direct access to the privileged vertebrate host blood compartment by infectious bite. Thus, hematophagous arthropods required new immune mechanisms to control the escape and dissemination of novel microbial pathogens from the midgut epithelial barrier. The APL1 gene family evolved specifically in mosquitoes, and APL1C is the paralog most related to the ancestral, single copy APL1 gene found in all examined *Anopheles* species [51]. The ancestral single-copy APL1, as found in *Anopheles* stephensi, displays an essential protective function against the enteric bacterial flora, with only a secondary effect on Plasmodium [36]. This APL1C-like ancestor expanded to three paralogs in a single lineage of African *Anopheles*, including the *A*. *gambiae/coluzzii* complex, and there the paralogs evolved new functional roles. The expanded paralogs in *A*. *gambiae/coluzzii* are also required for host protection against pathogenic fungi [52] and an *Anopheles*-transmitted arbovirus [53]. In these cases, it is not known if the protective activity is based on recognition and binding to virus particles or fungal cells, which would be surprising given the vastly different pathogen surfaces, but clearly APL1C (potentially in cooperation with other LRR proteins) displays a wider spectrum of immune protection capacities than merely against Plasmodium.

The current results raise multiple important questions that will require further work. First, what is the precise molecular target of APL1C binding, and what is the influence of the heteroduplex LRR protein partners on binding specificity? Mutagenized LRR proteins could be assayed in the *ex vivo* immune hemocoel system to measure differential ookinete binding efficiencies of different LRR homo- and heteroduplexes; a yeast display library of ookinete proteins could potentially identify ligands; and if successful, structural biology could reveal the basis of physical LRR interaction with ligands. Second, how is the pathogen specificity of LRR binding determined at the molecular level, such that APL1C can recognize and bind to both the ookinete and sporozoite stages of *P*. *berghei*, but not to the *P*. *falciparum* ookinete (nor presumably its sporozoites)? Finally, how is LRR binding translated into an appropriate effector response? The comparison of *P*. *berghei* ookinetes and sporozoites could be informative, because APL1C binds and functionally protects against both stages, but the protein partners including effectors are not shared. These and other questions will require considerable further work but can now be investigated.

The current results are consistent with and support the hypothesis that mosquito LRR proteins are functional counterparts of the immunoglobulin-based receptors that vertebrates use for discrimination of pathogens and targeting of immune effector activity. APL1C is one of the required components of a trimeric guard barrier that is best characterized for its protection against model rodent malaria parasites. The activity of the complex leads to the deployment of appropriate effector mechanisms directed against at least *P*. *berghei* ookinetes and sporozoites. APL1C also displays protective activity against diverse microbial targets, including at least an arbovirus and entomopathogenic fungus, but it is not yet known whether pathogen binding or a different mechanism underlie these interactions.

## Materials and methods

### Ethics statement

The protocol for the ethical treatment of the animals used in this study was approved by the research animal ethics committee of the Institut Pasteur, “C2EA-89 CETEA Institut Pasteur” as protocol number 202195.02. The Institut Pasteur ethics committee is authorized by the French Ministry of Higher Education and Research (MESR) under French law N° 2001–486, which is aligned with Directive 2010/63/EU of the European Commission on the protection of animals used for scientific purposes. The study was performed using practices and conditions approved by the Institut Pasteur Biosafety Committee as protocol number CHSCT 14.114.

### Mosquitoes and *Plasmodium* infection

The *Anopheles coluzzii* colony Fd03 initiated in Mali [54] and *A*. *coluzzii* Ngousso colony initiated in Cameroun [55] were reared at 26°C and 80% humidity, on a 12 h light/dark cycle with access to 10% sucrose. For infection with *Plasmodium berghei*, 3-week-old female SWISS mice were inoculated with 10^5^ erythrocytes infected with a strain of *P*. *berghei* expressing GFP under the control of the hsp70 promoter [56]. At 4 d post-injection, parasitemia was determined by flow cytometry of a tail blood sample, and male gametocyte maturity was verified by an exflagellation test [57]. Mice selected for mosquito infection had 4–8% parasitemia with mature gametocytes present. Mice were anaesthetized by intraperitoneal injection of ketamine at 125 mg/kg and xylazine at 12.5 mg/kg, mosquitoes were allowed to feed for 20 min, and only fully engorged females were used for further analysis. Mosquitoes were maintained at 21°C and 70% relative humidity with access to 10% sucrose. For infection with *Plasmodium falciparum*, *P*. *falciparum* strain NF54 was cultured using an automated tipper-table system [58] as implemented in the CEPIA mosquito infection facility of the Institut Pasteur [38]. Briefly, *A*. *coluzzii* line Fd03 mosquitoes were fed on mature gametocytes mixed with fresh erythrocytes in AB+ human serum in a water-jacketed membrane feeder at 37°C for 15 min, and only fully engorged females were used for further analysis. Mosquitoes were maintained at 26°C and 70% relative humidity with access to 10% sucrose.

### Protein purification and analysis

For hemolymph perfusion, groups of 10 female mosquitoes were rinsed once in ethanol and twice in PBS and placed on a glass slide. The two terminal segments of the abdomen were removed with a scalpel, 10 μL of PBS were injected into the thorax, and the flow-through was collected in a single microfuge tube as a pool for the 10 mosquitoes. For whole-mosquito protein purification, pools of 8 females were placed in a tube with glass beads (1 mm, Fisher Scientific) and 150 μl of 1X RIPA buffer (Cell Signaling Technology) with 1X protease inhibitor cocktail (Roche Life Science). Samples were homogenized using a FastPrep96 shaker (MP Biomedicals) using 3 cycles of 1400 rpm shaking for 30 s each. Homogenized samples were centrifuged for 5 min, 18000 g, 4°C. Collected supernatant was sonicated on ice and centrifuged for 15 min, 18000 g, 4°C.

For electrophoretic analysis, samples in XT sample buffer (Bio-Rad Laboratories), 1X DTT (Sigma Aldrich) were reduced by heating at 95°C for 5 min. Samples were separated on 4–12% Criterion SDS-PAGE gels (Bio-Rad Laboratories). Proteins were transferred to a nitrocellulose membrane using the Trans-Blot Turbo Transfer System (Bio-Rad Laboratories). Blots were blocked for 1 h in 5% bovine serum albumin (BSA, Sigma Aldrich) in Tris-buffered saline (Sigma Aldrich), 0.1% Tween 20 (TBST, Sigma Aldrich). To detect APL1C protein, immunoblots were probed overnight with a rabbit anti-APLC antibody [29] (1:5000 in TBST), rinsed three times in TBST for 10 min each, followed by 1 h with horseradish peroxidase-conjugated (HRP) anti-rabbit IgG secondary antibody (Cell Signaling Technology, 1:10000 in TBST). A mouse anti-GADPH antibody (Invitrogen, 1:4000 in TBST), followed by HRP anti-mouse IgG (H+L) secondary antibody (Thermofisher, 1:8000 in TBST) was used as a loading control. To detect V5-tagged proteins in 4a3A cells, immunoblots were probed with a mouse monoclonal antibody (mAb) anti-V5 at 1:5000, followed by 1 h with HRP-conjugated anti-mouse IgG secondary antibody (Promega France) at 1:10000. To detect TEP1 protein, immunoblots were probed with a rabbit anti-TEP1-C antibody (obtained from Elena Levashina, Max Planck Institute) at 1:100 and a mouse anti-tubulin mAb at 1:5000 (Sigma Aldrich), followed by 1 h with HRP-conjugated anti-rabbit or anti-mouse IgG secondary antibodies (Promega France) at 1:10000. For the APL1C time course shown in Fig 6A, the GADPH loading control normalized APL1C protein signal densities were compared to unfed (UF) controls, defined as 100%. Detection for all immunoblots was performed using the Enhanced Chemiluminescence system (Bio-Rad Laboratories) following the manufacturer’s instructions.

Immunoblot signals were quantified using Image Lab analysis software (Bio-Rad Laboratories). For APL1C levels in hemolymph after non-infective bloodmeal (NBM) and in unfed mosquitoes (UF), the specific densitometry of each band was determined as adjusted volume, by subtracting the area containing the band by an adjacent empty area. Relative quantity of APL1C level is the ratio of NBM/UF adjusted volumes. The levels of APL1C in whole mosquitoes were first normalized against the GADPH control and then calculated as NBM/UF ratios. For the levels of TEP1 in 4a3A cells, the ratio of TEP1/tubulin signals was calculated and the percentage of TEP1 signal reduction relative to tubulin was quantified in dsTEP1-treated cells as compared to dsGFP treated controls.

### Confocal immunofluorescence assay (IFA) and image analysis

Immunostaining of dissected midguts was performed as previously described [59]. Briefly, midguts were dissected in sterile 1× PBS, fixed for 40 s in 4% paraformaldehyde (PFA, Electron Microscopy Sciences), and were cut open longitudinally in sterile PBS to remove the blood bolus. Cleaned midguts were then fixed in 4% PFA in 1× PBS for 1 h at room temperature and rinsed five times in sterile PBS for 5 min each. Midguts were then blocked in 1% BSA for 2 h at room temperature. For detection of APL1C, midguts were incubated overnight with rabbit anti-APL1C antibody (1:600 in 1% BSA) at 4°C. Midguts were then rinsed five times for 10 min each with 1% BSA, followed by a 2 h incubation at room temperature with Alexa Fluor 647 anti-rabbit IgG (H+L) secondary antibody (Invitrogen, 1:500 in 1% BSA), and rinsed five times in 1% BSA, 10 min each. For detection of LRIM1, midguts were incubated overnight with mouse anti-LRIM1 mAb (1:10), obtained from Eric Marois, Institute of Molecular and Cell Biology, Strasbourg, and visualized by Alexa Fluor 555 anti-mouse IgG (H+L) secondary antibody (Invitrogen, 1:500). For detection of *P*. *falciparum* and *P*. *berghei* ookinetes, midguts were immunostained using mouse anti-Pfs25 antibody (1:10) or anti-Pys25 mAb (1:100), obtained from Chris Janse, Leiden University Medical Center. The Pys25 antibody identified using *P*. *yoelii* cross-reacts with the *P*. *berghei* Pbs25 ortholog [60]. For midgut permeabilization, 1% BSA was supplemented with 0.1% Triton X-100 (Sigma Aldrich). The permeabilization control to confirm antibody accessibility within the midgut tissue was performed using anti-GFP, rabbit polyclonal antibody, Alexa Fluor 647 conjugate, at a concentration of 10 μg/ml (Invitrogen). Immunostained tissues were counterstained for actin with 405 phalloidin-iFluor (Abcam, 1:1000) for 10 min and subsequently for nuclei with DAPI (Thermo Fisher Scientific, 10 μg/ml,) with 12 rpm rocking for 10 min. Tissues were rinsed in sterile PBS and mounted in Vectashield (Vector Laboratories).

For quantification of APL1C abundance in fixed midguts, samples were imaged on a laser-scanning confocal microscope (LSM700, Carl Zeiss Jena). All acquisitions were configured as follows: pinhole size 1 Airy unit; DAPI/Phalloidin channel: 405 nm laser, SP490 filter; APL1C channel: LP640 filter, laser 633 nm; images are 1024 x 1024, digitized over 16-bit; Z step interval: 0.33 μm; objective 40x, oil, NA = 1.3, Plan-Neofluar. Images were analyzed with Fiji [61]. For APL1C signal quantification, 3D Images were filtered first with a 3x3 median filter then with a Gaussian filter with sigma = 0.7 pixels. The 3D stacks were then projected using Maximum Intensity Projection. A selection was created using an intensity threshold using ImageJ ‘default’ method. Reported measurements represent the mean intensity for the channel APL1C in the selection. Ookinete microscopy used a high-speed spinning-disk confocal system UltraVIEW ERS (Perkin Elmer) mounted on an inverted Axiovert 200 microscope (Carl Zeiss). All images used for APL1C abundance were acquired and analyzed using the same parameters.

For the dextran injection control, cold-anesthetized *A*. *coluzzii* females were injected intrathoracically with 138 nl of 10 mg/ml of CF488A Dye Dextran 70,000 MW (Biotium) using a glass capillary needle and a Nanoject II injector (Drummond Scientific). At 1.5 h post-injection, midguts were dissected, fixed and counterstained with DAPI and phalloidin as described above.

For microbiome analysis, adult mosquitoes were fed on cotton soaked with 10% sucrose with antibiotics (ATB) penicillin 62.5 μg/mL, streptomycin 100 μg/mL and gentamicin 50 μg/mL (Dominique Dutcher SAS). Mosquitoes were bloodfed on a mouse for 20 min and unfed mosquitoes were discarded. ATB treatment was continued after bloodfeeding. At 24 h post-bloodmeal, midguts were dissected from antibiotic treated and control mosquitoes and subject to confocal IFA detection of APL1C as above. Antibiotic effectiveness was confirmed 24 h post-bloodmeal by quantifying the total bacterial load using quantitative PCR (qPCR) of the bacterial 16S ribosomal RNA gene as described [36]. Briefly, mosquitoes were washed in 75% ethanol and then sterile PBS, midguts were dissected from antibiotic treated and control mosquitoes, and DNA was extracted with DNeasy PowerSoil Kit (QIAGEN GmbH). The V4 region of the 16S rDNA (S4 Table) was used for qPCR. DNA samples from each independent biological replicate were used to perform distinct qPCR in triplicate and fold changes obtained between (+ATB) and (-ATB) were combined as a mean.

### *Ex vivo Anopheles* hemocoel system

*A*. *coluzzii* 4a3A hemocyte-like cells were cultured in monolayer at 27°C in Insect Xpress medium (Lonza Group) supplemented with 5% fetal bovine serum (GIBCO BRL) and 50 μg/ml gentamycin (Sigma Aldrich). Before transfection, 1 x 10^5^ cells were incubated in an 8-well Lab-Tek chamber slide (Thermo Fisher Scientific) for 1 h. Cells were transfected with the relevant expression vector using Cellfectin II reagent (Invitrogen) according to the manufacturer’s protocol.

Plasmids expressing tagged APL1C and LRIM1 proteins were constructed from genes amplified from DNA of *A*. *coluzzii* Ngousso mosquitoes using primers flanking the coding regions of each gene (S3 Table) and were cloned into a dual His- and V5-tag insect expression vector (pAc5.1 V5-His, Invitrogen). To generate a control plasmid expressing the irrelevant protein for Red Fluorescent Protein (RFP), the coding sequence from the mammalian expression vector pTagRFP (Evrogen) was cloned in the pAc5.1 V5-His expression vector using EcoRI and NotI restriction enzymes. To promote RFP protein secretion from cells, the signal sequence from APL1C was amplified from *A*. *coluzzii* Ngousso mosquitoes with primers (S3 Table) and cloned in the pAc5.1 sRFP-V5 expression vector.

Midguts of *A*. *coluzzii* Ngousso mosquitoes infected with *P*. *berghei* were dissected at 24 h post-infection into PBS and placed directly for 2 h at 21°C in culture medium of 4a3A cells that were transfected 3 days before with pAc5.1 sRFP-V5, pAc5.1 APL1C-V5 or pAc5.1 LRIM1-V5 expression vectors. After incubation, transfected cells and dissected midguts were treated for immunostaining analysis with a mouse anti-V5 mAb (Invitrogen, 1:500), followed by 1 h with Alexa Fluor 594 anti-mouse IgG (H+L) secondary antibody (1:2000). As a control to detect extracellular ookinetes, midguts were incubated with a mouse mAb anti-Pbs28 antibody [39] at 1/500 and detected as above. Nuclei were stained with Hoechst 33342. After mounting in SlowFade Gold antifade reagent (Molecular Probes), samples were observed using a Leica DM 5000 B fluorescent microscope.

### RNAi-mediated gene silencing

Double-stranded RNA (dsRNA) was synthesized from PCR amplicons using the T7 Megascript Kit (Ambion, Inc). Primers sequences are listed in S4 Table. For gene silencing in 4a3A cells, 500 ng of dsTEP1 was transfected into a confluent culture of 1 x 10^5^ 4a3A cells using Cellfectin II reagent (Invitrogen). After 3 days, the conditioned media were exchanged for a fresh medium and cells were transfected with the expression vector after which 500 ng of dsRNA were added again. The efficiency of gene silencing effect was monitored at day 6, prior to introduction of *P*. *berghei*-infected midguts into the *ex vivo* hemocoel assay, by western blotting analysis of cells and culture medium protein extract using rabbit anti-TEP1 antibody (obtained from Elena Levashina, Max Planck Institute) and a mouse anti-tubulin antibody (Sigma). For *in vivo* gene silencing in mosquitoes, 500 ng of dsRNA was injected into the thorax of cold-anesthetized *A*. *coluzzii* females using a glass capillary needle and Nanoject II injector (Drummond Scientific). The efficiency of gene silencing in mosquitoes was monitored at 3d post-injection by reverse transcriptase quantitative real-time PCR (RT-qPCR) of mosquito RNA as described [16], using SYBR Green Supermix (KAPA SYBR FAST ABI, Kapa Biosystems) and the CFX96 Touch Real-Time PCR Detection System (Bio-Rad Laboratories). Total RNA was extracted with TRI Reagent (Molecular Research Center, Inc.) and reverse transcribed to cDNA using M-MLV reverse transcriptase (Invitrogen). Primers are listed in S4 Table. The gene for ribosomal protein S7 (rpS7) was used as an internal control. The quantification of each gene was a ratio to rpS7. Analysis of transcript abundance relative to rpS7 was determined according to the 2−ΔΔCt method [62]. PCR cycling conditions were: 95°C for 10min, 40 cycles of [95°C for 15 sec, 60°C for 1 min].

### Clodronate depletion of phagocytic hemocytes

Phagocyte depletion using clodronate liposomes (CLD) was performed as previously described [37]. Briefly, female mosquitoes were injected intrathoracically with 101.2 nl of CLD at a concentration of 1.25 mg/ml (Standard Macrophage Depletion Kit; Encapsula NanoSciences) using a Nanoject II injector (Drummond Scientific), or with the same amount of control non-clodronate liposomes (LPs). Injections were performed 24 h before mosquito infection by feeding on a *P*. *berghei*-infected mouse. The efficiency of phagocytic cell depletion by CLD was tested by measuring the expression level of two phagocytic cell markers, eater and nirmrodB2, by RT-qPCR of pools of 8 CLD and LP-injected mosquitoes.

### Sporozoite quantification in salivary glands

Salivary glands were collected from mosquitoes infected with GFP-expressing *P*. *berghei* at 19 d post infection essentially as previously reported [63]. Briefly, mosquitoes were rinsed one time in ethanol and two times in PBS, placed on a glass side in a drop of PBS, and salivary glands were extracted from the thorax under a stereomicroscope using two needles of insulin syringes. Salivary glands were placed in a 1.5-ml microcentrifuge tube containing 20 μl of PBS and placed on ice. The pair of salivary glands from a single mosquito was collected into the same tube and gently crushed with a microfuge pestle to release sporozoites. The solution was homogenized by pipetting, sporozoites were centrifuged through a 35 μm filter (BD Falcon, BD Biosciences) to remove remaining salivary glands debris, and 10 μl of the solution was placed in a Kova Glasstic Slide 10 with Grids (Kova International). Sporozoites were allowed to settle for 30 min and were counted with an epifluorescence microscope using a GFP filter.

### Sporozoite counting and immunostaining

For flow cytometry analysis, GFP-expressing *P*. *berghei* hemolymph sporozoites were counted originating from a single infected mosquito subjected to dsAPL1C, dsLRIM1 or dsGFP treatment. Individual mosquitoes were perfused using 200 μl PBS per individual mosquito. For APL1C binding to the sporozoites, GFP-expressing *P*. *berghei* hemolymph sporozoites were perfused from mosquitoes in 4% PFA. Sporozoites were washed with 700 μl PBS, centrifuged to pellet, resuspended in anti-APL1C antibody diluted in 1% BSA (1:300), and incubated on ice for 1 h followed by 30 min incubation on ice with Alexa Fluor 555 anti-rabbit IgG (H+L) secondary antibody in 1% BSA (Invitrogen, 1:500). Sporozoite samples were diluted in 200 μl PBS and loaded into a CytoFLEX S flow cytometer (Beckman Coulter), gated in a side scatter height (SSC-H) versus GFP dot plot graph. Gating of the GFP-expressing sporozoite population was confirmed by CSP staining with a primary mouse anti-CSP antibody [64] and Alexa Fluor 647 anti-mouse IgG (H+L) secondary antibody (Invitrogen) in a GFP versus CSP dot plot graph (S12B Fig). It is known that injection of mosquitoes with control dsGFP RNA does not influence GFP fluorescence of expressing parasites infecting the mosquitoes [65]. For APL1C gating, the fluorescence corresponding to APL1C staining was determined from the GFP population. In order to determine APL1C signal threshold, sporozoites were incubated with an antibody different from anti-APL1C, specifically mouse anti-CSP, and Alexa Fluor 555 anti-rabbit IgG (H+L) secondary antibody. Data were analyzed with CytExpert 2.0 or FlowJo 10.2 software.

For IFA analysis, infected mosquitoes were perfused using 4% PFA into an Ibidi chamber slide (Ibidi GmbH, Germany). Perfused sporozoites were centrifuged for 2 min at 500 g and left for 30 min at room temperature to fix and sediment on the bottom of the chamber. After incubation, sporozoites were centrifuged for 2 min, 500 g. The chamber was rinsed 3 times with PBS. Next, sporozoites were incubated for 1 h in 1% BSA at room temperature and subsequently with anti-APL1C antibody diluted in 1% BSA (1:300) overnight at 4°C. The next day, sporozoites were rinsed three times for 10 min each with 1% BSA, followed by a 2 h incubation at room temperature with Alexa Fluor 647 anti-rabbit IgG (H+L) secondary antibody (Invitrogen, 1:500 in 1% BSA). Then, sporozoites were rinsed three times for 10 min each in 1% BSA, and counterstained for 10 min with DAPI. All incubations were performed with 12 rpm rocking. Sporozoites were rinsed in sterile PBS and observed under a fluorescent microscope.

### Statistical analyses

For western blot analysis, normalized band intensity was compared by unpaired t-test. Differences in APL1C protein abundance by confocal IFA of midguts and differences in perfused sporozoite numbers were tested using Wilcoxon–Mann–Whitney test. Abundance differences were first tested independently for each replicate (individual p-values in S1 Table), and if individual replicates showed a common trend of change, individual p-values were combined using the meta-analytical approach of Fisher [66]. For RT-qPCR analysis of RNA transcript levels relative to rpS7, the 2−ΔΔCt method was used, and difference in deltaCt distribution across biological replicates was statistically tested using unpaired t-test. Differences in salivary gland infection prevalence were tested using the chi-square test. Infection differences were first tested independently for each replicate (individual p-values in S2 Table), and if individual replicates showed a common trend of change, individual p-values were combined using the meta-analytical approach of Fisher. Data were analyzed using GraphPad Prism 8.0

## Supporting information

S1 Fig Uninfected bloodmeal induces hemolymph APL1C protein. **A**. Immunoblot analysis of APL1C protein level in hemolymph after NBM. Mosquito hemolymph was collected at 24 h post-NBM and analyzed by western blot using APL1C antibody in three biological replicates, with hemolymph from UF mosquitoes as the control. The APL1C protein bands quantified as a unit are indicated by the red bracket, protein size ladder (kD) shown in green on the left of protein samples. **B**. Hemolymph APL1C levels quantified by densitometry of western blots. First, for each sample, the specific APL1C signal was determined by subtracting the background signal of an adjacent same-size empty area (adjusted volume intensity, Adj. Vol. Int.). Secondly, the NBM/UF ratio of APL1C protein levels (Rel. Quant,) was calculated for each biological replicate. **C**. The XZ and YZ orthogonal confocal views of the NBM stack picture show that APL1C protein (red) localizes extracellularly, on the basal side (ba) and not lumen side (lu) of the midgut surface. **D**. Midgut basal lamina is permeable to a control molecule of similar molecular mass to APL1C protein. Confocal imaging analysis of midguts from mosquitoes injected with 70 kDa fluorescent dextran polymer indicates that dextran (red) diffuses through basal lamina and is captured on fixed midguts, with an appearance to immunostained APL1C on fixed midguts. For C and D nuclei and actin were stained with DAPI and Phalloidin (blue), respectively and the scale bar is 10 μm.
(PDF)

S2 Fig APL1C antibody signal in fixed midguts is specific to the APL1C protein. **A.** Efficient APL1C silencing by dsAPL1C treatment was verified by RT-qPCR comparison of dsAPL1C and control dsGFP treated mosquitoes (dotted line, dsGFP) 72 h post-injection. The ratio of normalized APL1C transcript in dsAPL1C relative to dsGFP treatments was calculated using triplicates from the same cDNA dilution. Graph represents mean with ±SEM of the expression fold change between “dsAPL1C” and “dsGFP” control from two biological replicates (N = 2). **B.** APL1C gene silencing abolishes APL1C signal detected by immunostaining with anti-APL1C antibody of NBM mosquito midguts. The APL1C confocal IFA signal used on fixed midguts to quantify relative abundance of APL1C is observed on the midguts from dsGFP-injected control mosquitoes (red). Nuclei and actin were stained with DAPI and Phalloidin (blue). The scale bar is 10 μm.
(PDF)

S3 Fig Bloodmeal induction of hemolymph APL1C is not dependent upon proliferation of the enteric microbiome. **A**. Bacterial abundance in mosquitoes was significantly reduced after antibiotic treatment, as confirmed by qPCR quantification of 16S ribosomal gene DNA (16S rDNA) in mosquitoes exposed (+ATB) or not (control, -ATB, depicted as a dotted line) to antibiotics at 24 h post-NBM. The ratio of normalized 16S rDNA detection in “+ATB” versus “-ATB” treatments was calculated using triplicates from the same cDNA dilution. Graph represents mean with ±SEM of the fold change between +ATB and -ATB from two biological replicates (N = 2). **B.** Immunostaining analysis of +ATB midguts indicates that bacterial depletion did not alter APL1C protein localization on NBM midguts. Images are representative of two independent biological replicates (N = 2, 3–7 midguts per experimental point). The scale bar is 10 μm.
(PDF)

S4 Fig APL1C protein is localized in hemocyte vesicles. Liposome-encapsulated clodronate injection depletes mosquito phagocytic cell markers. **A.** Immunostaining analysis of perfused hemocytes and **B** cultured 4a3A cells indicates that APL1C protein is localized in vesicles or vesicle-like structures (red). Cells were stained with DAPI to label nuclei (blue) and phalloidin to label actin (green). Bright field indicated as BF. The scale bar is 5 μm. **C.** Clodronate-mediated phagocytic cell depletion was verified by the qPCR measurement of phagocytosis markers eater and nimrodB2 between mosquitoes injected with clodronate (CLD) and control empty liposomes (LP, dotted line) at 24 h post-injection. The ratio of normalized eater or nimrodB2 cDNA detection in CLD and LP treatments was calculated using triplicates from the same cDNA dilution. Graph represents mean with ±SEM of the expression fold change between CLD and LP from three biological replicates (N = 3). Data for qPCR analysis was analyzed by unpaired t-test (significance levels of t-test p-values: * p-value<0.05; ** p-value <0.01).
(PDF)

S5 Fig APL1C binds to extracellular *P*. *berghei* ookinetes. XZ and YZ orthogonal views of the confocal stack images link APL1C protein binding with parasite spatial localization in mosquito midguts. Yellow lines depict the location of the parasite, for which spatial localization is presented on the sides of the stack picture. Orientation of the midgut epithelium (lu, lumen side, ba, basolateral side) is indicated by labeled arrows on the upper left panel and applies to all panels shown. Parasites in the panels bounded by the red line were external to the basolateral side of the midgut and are labeled by APL1C protein (APL1C, red; GFP, green). Parasites in the panels bounded by the green line remained in the lumen or within epithelial cells of the mosquito midgut are not labelled with APL1C (GFP, green). The scale bar is 5 μm.
(PDF)

S6 Fig Midgut permeabilization enables antibody access to *P*. *berghei* parasites in all midgut locations. IFA of midguts collected 24 h post-IBM. Midguts were permeabilized and immunostained with anti-GFP conjugated antibody. GFP-expressing ookinetes (green) were also associated with anti-GFP antibody (red) which confirmed antibody accessibility to all parasites in mosquito midguts. Nuclei and actin were stained with DAPI and Phalloidin (blue). The scale bar is 10 μm.
(PDF)

S7 Fig V5-tagged protein constructs are secreted from the hemocyte-like 4a3A cell line. **A.** Immunoblot analysis of culture medium (M) and cells (C) of 4a3A cells transfected with plasmids encoding V5-tagged RFP (RFP-V5) and V5-tagged RFP fused with the signal sequence from APL1C (sRFP-V5). Immunoblot was probed with anti-V5 antibody. **B.** Immunoblot analysis of culture medium of 4a3A cells transfected with plasmids encoding V5-tagged APL1C and LRIM1 under reducing (R) and non-reducing (NR) conditions with anti-V5 antibody. Estimated sizes of monomeric APL1C and LRIM1 forms including V5-tag are: 88 kDa (APL1C) and 60 kDa (LRIM1), respectively. The results indicate that both APL1C-V5 and LRIM1-V5 are secreted into the culture medium.
(PDF)

S8 Fig Both APL1C and LRIM1 can co-bind to the same individual *P*. *berghei* ookinetes. Immunostaining analysis of non-permeabilized *P*. *berghei*-infected mosquito midguts 24 h post-infection. Live, GFP-expressing (green) parasites were tested for APL1C (red) and LRIM1 (cyan) protein binding by incubation with rabbit-originated APL1C and mouse-originated LRIM1 antibodies and different fluorophore-conjugated species-specific secondary antibodies. Images shown of APL1C-positive LRIM1-positive parasites are representative of two biological replicates (N = 2). Nuclei and actin were stained with DAPI and Phalloidin (blue). The scale bar is 2 μm.
(PDF)

S9 Fig TEP1 protein is efficiently depleted by gene silencing in 4a3A cells. **A.** Western blot analysis of TEP1 protein in the 4a3A cell line. The efficiency of TEP1 gene silencing was monitored 6 d after treatment with dsTEP1 or dsGFP in cells transfected with the constructs APL1C-V5, LRIM1-V5 or both. Detection used anti-TEP1 antibody with anti-alpha tubulin antibody as a loading control. **B.** Quantitative analysis of TEP1 protein immunoblotting. Expression ratio of TEP1 and tubulin loading control protein levels were quantified by densitometry. For each condition, the ratio of protein levels TEP1/tubulin was calculated and the percentage of TEP1 signal reduction relative to tubulin was quantified in cells treated with dsTEP1 as compared to dsGFP.
(PDF)

S10 Fig APL1C binding to extracellular *Plasmodium berghei* ookinetes and APL1C functional immune protection are not influenced by infection intensity. **A.** Immunostaining of a non-permeabilized midgut from a *P*. *berghei* low intensity infection (<10 ookinetes per midgut) 24 h post-infection detects APL1C protein binding to extracellular ookinetes. Extracellular location of parasites is indicated by staining with antibody directed against Pys25 ookinete surface protein (yellow), APL1C binding is indicated by anti-APL1C antibody staining (red). Nuclei and actin were stained with DAPI and phalloidin, respectively (blue). Scale bar, 10 μm. **B.** Pie chart depicts the APL1C binding outcome for all extracellular (Pys25-positive) parasites in 12 midguts with low intensity (<10 ookinetes per midgut) *P*. *berghei* infections (red slice, APL1C-positive extracellular ookinetes, green pie slice, APL1C-negative extracellular parasites). The 12 midguts infected with less than 10 ookinetes per midgut carried 36 total extracellular ookinetes of which 23 (63%) were APL1C-positive. n indicates number of live and dead ookinetes labelled with antibody against Pys25 ookinete surface protein. **C. D.** APL1C expression was silenced in mosquitoes infected with *P*. *berghei* at low oocyst infection prevalence (<35% infected mosquitoes in dsGFP control) and intensity (median = 2 oocysts in dsGFP control) **C.** Pie charts indicate that APL1C silencing causes higher infection prevalence as compared to dsGFP controls. n is the total number of dissected mosquitoes from the two replicates. **D.** Plot indicates that APL1C silencing causes higher infection intensity (median = 9 oocysts) as compared to dsGFP controls (median = 2 oocysts). Combined p-values from two independent replicates (N = 2) are obtained using the Fisher method; **** indicate P-value<0.0001.
(PDF)

S11 Fig APL1C protein abundance in response to bloodmeal and *P*. *berghei* parasite development. **A.** Western blot analysis of APL1C protein levels after IBM and NBM. Mosquitoes were collected at 4 d (early oocyst), 10 d (mature oocyst before sporozoites release), 12 d (early sporozoite release) and 18 d (late sporozoite release) and analyzed by western blot using anti-APL1C antibody and anti-GADPH antibody as a loading control. NBM and UF mosquitoes from corresponding time-points were used as controls. Depicted blot is representative of three independent replicates. **B.** Loading-control normalized APL1C protein band density ratios of IBM/UF and IBM/UF were calculated for each time point in each biological replicate.
(PDF)

S12 Fig APL1C gene silencing depletes transcript and protein for at least 10 days. **A.** APL1C expression is reduced at the time of salivary gland dissections done at 10 d post dsAPL1C injection. APL1C silencing was verified by qPCR measurement between dsAPL1C and dsGFP (dotted line) treatments 10 d post-treatment. The ratio of normalized APL1C transcript in dsAPL1C versus dsGFP treatments was calculated using triplicates from the same cDNA dilution. Graph represents mean with ±SEM of the expression fold change between dsAPL1C and dsGFP control from four biological replicates (N = 4). Data for qPCR analysis was analyzed by unpaired t-test (significance level of t-test **** p-value <0.0001). **B.** Western blot analysis of APL1C protein level in mosquito 10 d after APL1C gene silencing. The efficiency of APL1C depletion was monitored at day 10 post dsAPL1C injection versus dsGFP control in whole mosquitoes using anti-APL1C antibody and anti-GADPH antibody as a loading control. **C.** APL1C protein level is still decreased 10 d after dsAPL1C injection. The loading control normalized APL1C protein band densities were compared between dsAPL1C and dsGFP (dotted line) injected mosquitoes.
(PDF)

S13 Fig Flow cytometric analysis of APL1C binding to *P*. *berghei* sporozoites *in vivo*. **A**. GFP-expressing sporozoites perfused in mosquito hemolymph were gated in a side scatter height (SSC-H) versus GFP in a dot plot graph. **B**. The sporozoite population that expressed GFP was confirmed by CSP staining, which yielded the same number of events. **C**. APL1C gating strategy. The fluorescence corresponding to APL1C staining was determined from the GFP population. **D**. APL1C gating control to determine APL1C signal threshold. Sporozoites were incubated with an antibody different from anti-APL1C (mouse anti-CSP) and Alexa Fluor 555-conjugated anti-rabbit secondary antibody. **E**. APL1C labelling of hemolymph perfused sporozoites, at 12 d, 15 d and 22 d post mosquito infection. Sporozoites in each replicate are reported as percentage [%] or number of APL1C labelled sporozoites out of global sporozoites population per each collection timepoint.
(PDF)

S14 Fig APL1C antibody labelling of P. berghei sporozoites is specific to the APL1C protein. **A.** Sporozoites perfused from control dsGFP-treated mosquitoes are labelled by APL1C protein. The top panel depicts merged images of representative sporozoites (scale bar 10 μm), whereas the images below show 3 channels of enlarged projections of sporozoites (scale bar 2 μm), indicated by the numbers on the merged image. **B.** Sporozoites perfused from dsAPL1C-treated mosquitoes are not labelled by APL1C protein. The top panel depicts merged images of representative sporozoites (scale bar 10 μm), whereas the images below show 3 channels of enlarged projections of sporozoites (scale bar 2 μm), indicated by the numbers on the merge picture.
(PDF)

S15 Fig Activity of APL1C and LRIM1 do not directly reduce P. berghei sporozoite numbers in the mosquito hemocoel. **A.** APL1C was silenced by dsRNA treatment of mosquitoes at 9 d post-IBM, prior to sporozoite release from oocysts. At 17 d post-IBM, during sporozoite release, mosquitoes were perfused individually and the number of circulating sporozoites per individual mosquito was counted by flow cytometry in mosquitoes treated with dsAPL1C or control dsGFP. Graph presents the sporozoite number in perfused individuals between dsAPL1C- and dsGFP-treated mosquitoes. Each point represents a single perfused individual, bars represent mean with ±SEM. Sample sizes (N) show the number of independent replicate experiments, (n) the total number of perfused individuals across replicates. Data were compared between the two conditions by Mann-Whitney test. All statistical differences tested independently within replicates (individual p-values in S1 Table) (significance level of Mann-Whitney n.s. = not significant). **B.** APL1C silencing by dsAPL1C treatment 9 d post-IBM was still efficient at the time of mosquito perfusion, 17 d post-IBM, as verified by qPCR. The ratio of the normalized APL1C cDNA detection in dsAPL1C versus dsGFP treatments was calculated using triplicates from the same cDNA dilution. Graph represents mean with ±SEM of the transcript abundance fold change between dsAPL1C and dsGFP samples from independent biological replicates (N). Data for qPCR analysis was analyzed by unpaired t-test (significance levels of t-test p-values: *** p-value <0.001). **C.** Test of LRIM1 function for hemocoel sporozoite numbers. Description as in panel A but substituting dsLRIM1 in place of dsAPL1C. **D.** LRIM1 silencing by dsLRIM1 treatment 9 d post-IBM was still efficient at the time of mosquito perfusion, 17 d post-IBM, as verified by qPCR. Description as in panel B but substituting dsLRIM1 in place of dsAPL1C.
(PDF)

S16 Fig Activity of TEP1 and TEP3 are not protective against *P*. *berghei* sporozoites. **A.** TEP1 and TEP3 expression is efficiently silenced at the time of salivary gland dissection (10 d post-dsTEP1, dsTEP3 or dsTEP1/TEP3 injections). TEP1 and TEP3 silencing was verified by the qPCR measurement. The ratio of the normalized TEP1 or TEP3 cDNA detection in dsTEP1, dsTEP3 or dsTEP1/TEP3 versus dsGFP treatments was calculated using triplicates from the same cDNA dilution. Graphs represent mean with ±SEM of the expression fold change from independent biological replicates (N). Data for qPCR analysis was analyzed by unpaired t-test (significance levels of t-test p-values: ** p-value <0.01; *** p-value <0.001). **B.** TEP1, TEP3 or simultaneous TEP1/TEP3 depletion does not influence sporozoite salivary gland infection. The percentage of sporozoite-infected salivary glands (gray) or non-infected (white) in dsTEP1, dsTEP3 or dsTEP1/TEP3 mosquitoes and dsGFP injected control are shown in pie charts as the mean percentage obtained from independent number of biological replicates (N). Prevalence from each replicate was compared between the two conditions by chi-square test. All statistical differences were first tested independently within replicates (individual p-values in S2 Table), and if individual replicates showed a common trend of change, individual p-values were combined using the meta-analytical approach of Fisher (significance level of chi-square n.s. = not significant).
(PDF)

S1 Table Statistical analysis of APL1C abundance detected by confocal IFA on dissected fixed midguts. Summary data for all experimental replicates testing the APL1C protein signal between indicated treatments (Condition). The number of tested midguts in each treatment are indicated in the column titled, Number of midguts. Statistically significant differences are indicated by green shading. Row indicates the treatment for a given replicate, with the corresponding replicates indicated in the following row(s) for the statistical analysis. Individual p-values were calculated per replicate by statistical comparison to the corresponding control experiment shown in column 1 (Experiment #) and indicated in "Tested conditions" column. If the replicates of a tested condition were consistent (in the same phenotypic direction, see Methods), then the individual p-values were combined by Fisher’s method (Fisher combined prob). If the replicate phenotypes were not consistent, the individual p-values are shown but combining of p-values is not justified.
(XLSX)

S2 Table Statistical analysis of *P*. *berghei* salivary gland infection prevalence and intensity following gene silencing. Summary data for all experimental replicates testing the effect of targeted gene silencing compared to control treatment with dsGFP. Statistically significant differences are indicated by green shading. Row indicates targeted gene tested (injected dsRNA) for a given replicate, with the corresponding replicates indicated in the following row(s) for the tested targeted gene. Individual p-values were calculated per replicate by statistical comparison to the corresponding dsGFP control experiment shown in column 1 (experiment #). If the replicates of a tested gene were consistent (in the same phenotypic direction, see Methods), then the individual p-values were combined by Fisher’s method (Fisher combined prob). If the replicate phenotypes were not consistent, the individual p-values are shown but combining of p-values is not justified.
(XLSX)

S3 Table Primer sequences used for plasmid construction. (XLSX)

S4 Table Primer sequences used for dsRNA synthesis and qPCR. (XLSX)
