# Supplementary figures and images for: The Anopheles leucine-rich repeat protein APL1C is a pathogen binding factor recognizing Plasmodium ookinetes and sporozoites
Source: PLoS Pathog. 2024 Feb 14;20(2):e1012008. doi: 10.1371/journal.ppat.1012008 (PMC10898737; doi:10.1371/journal.ppat.1012008)

A

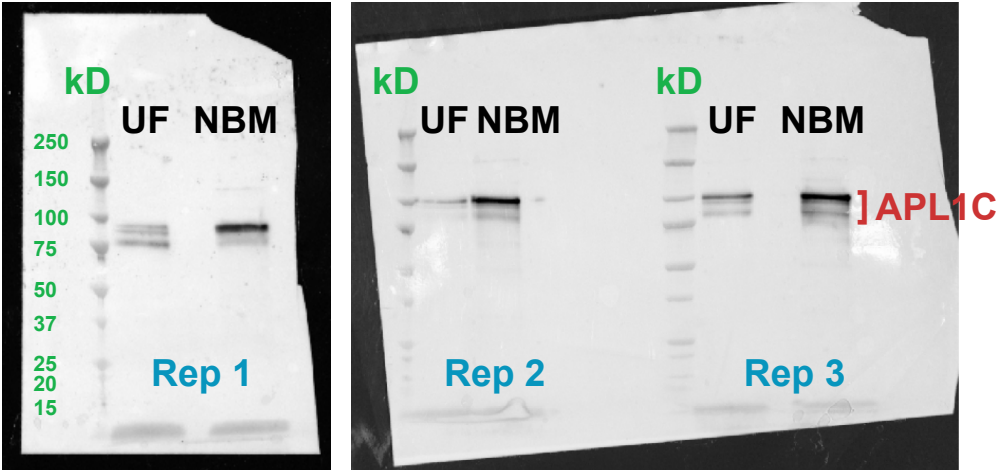

B

| Condition | Adj. Vol. (Int) |          |          | Rel. Quant. |       |       |
|-----------|-----------------|----------|----------|-------------|-------|-------|
|           | Rep 1           | Rep 2    | Rep 3    | Rep 1       | Rep 2 | Rep 3 |
| UF        | 1.55E+07        | 3.88E+06 | 8.89E+06 | 1.00        | 1.00  | 1.00  |
| NBM       | 3.15E+07        | 1.97E+07 | 2.40E+07 | 2.03        | 5.07  | 2.70  |

C

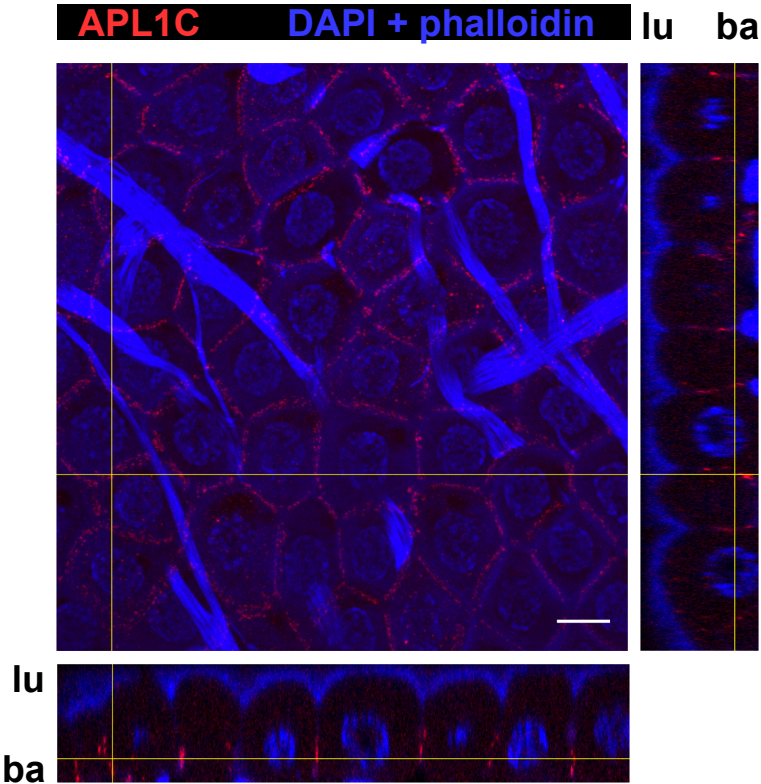

D

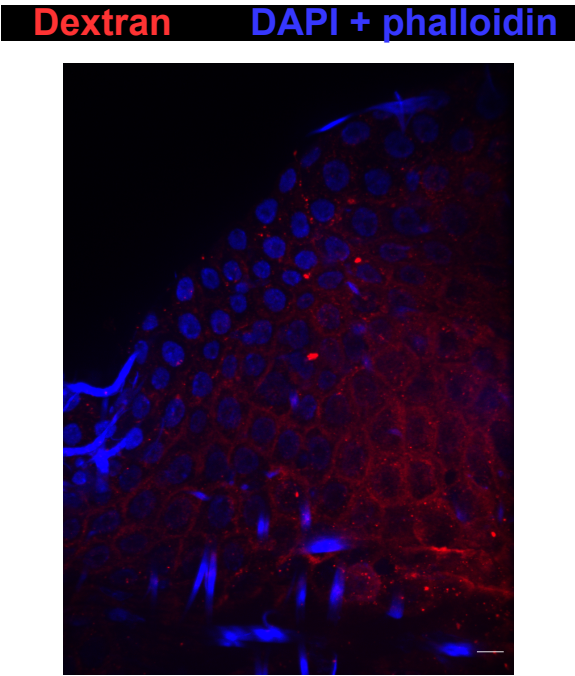

Supplement: S1 Fig — A. Immunoblot analysis of APL1C protein level in hemolymph after NBM. Mosquito hemolymph was collected at 24 h post-NBM and analyzed by western blot using APL1C antibody in three biological replicates, with hemolymph from UF mosquitoes as the control. The APL1C protein bands quantified as a unit are indicated by the red bracket, protein size ladder (kD) shown in green on the left of protein samples. B. Hemolymph APL1C levels quantified by densitometry of western blots. First, for each sample, the specific APL1C signal was determined by subtracting the background signal of an adjacent same-size empty area (adjusted volume intensity, Adj. Vol. Int.). Secondly, the NBM/UF ratio of APL1C protein levels (Rel. Quant,) was calculated for each biological replicate. C. The XZ and YZ orthogonal confocal views of the NBM stack picture show that APL1C protein (red) localizes extracellularly, on the basal side (ba) and not lumen side (lu) of the midgut surface. D. Midgut basal lamina is permeable to a control molecule of similar molecular mass to APL1C protein. Confocal imaging analysis of midguts from mosquitoes injected with 70 kDa fluorescent dextran polymer indicates that dextran (red) diffuses through basal lamina and is captured on fixed midguts, with an appearance to immunostained APL1C on fixed midguts. For C and D nuclei and actin were stained with DAPI and Phalloidin (blue), respectively and the scale bar is 10 μm. (PDF) [file ppat.1012008.s001.pdf]

**A**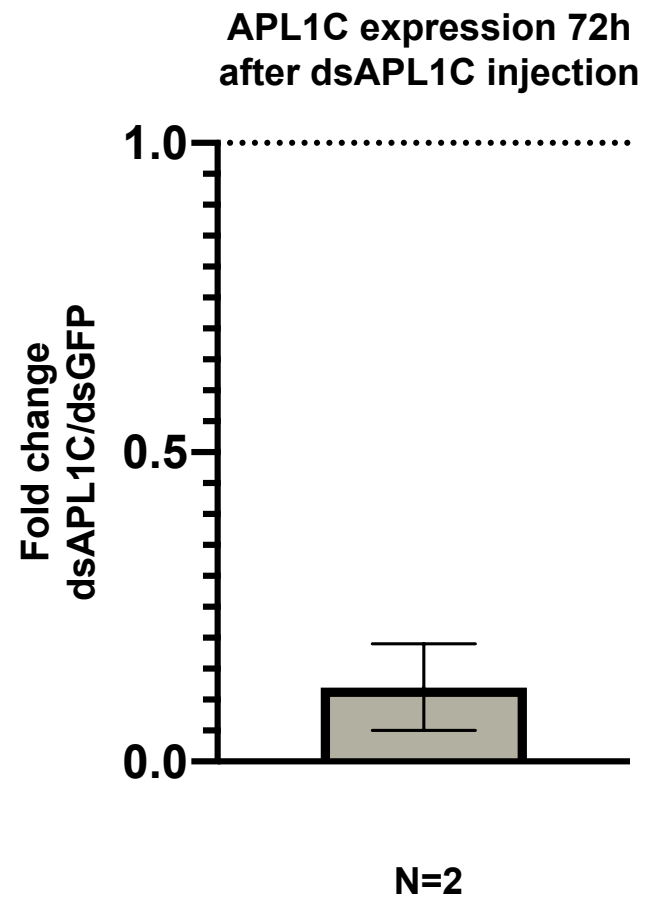**B**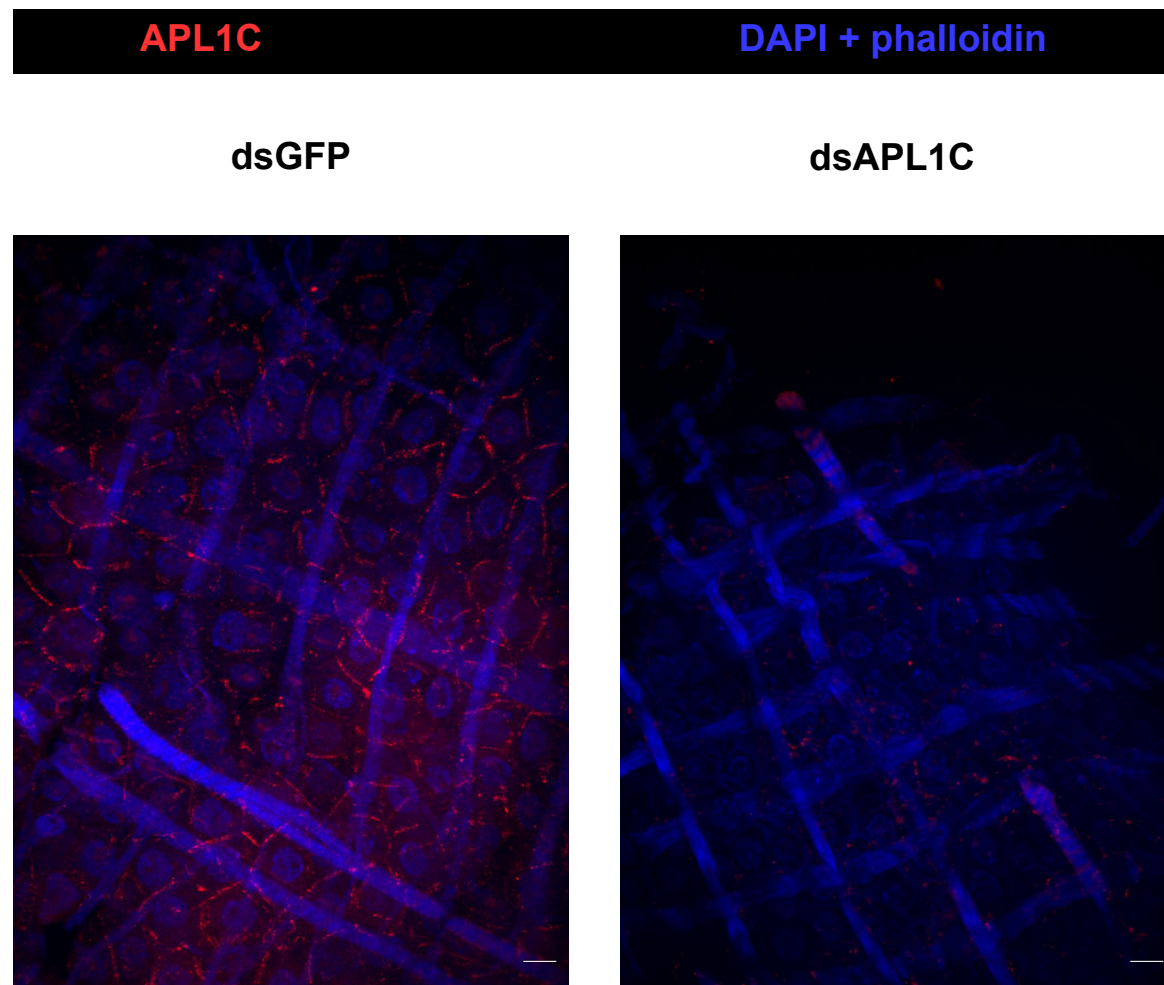

Supplement: S2 Fig — A. Efficient APL1C silencing by dsAPL1C treatment was verified by RT-qPCR comparison of dsAPL1C and control dsGFP treated mosquitoes (dotted line, dsGFP) 72 h post-injection. The ratio of normalized APL1C transcript in dsAPL1C relative to dsGFP treatments was calculated using triplicates from the same cDNA dilution. Graph represents mean with ±SEM of the expression fold change between “dsAPL1C” and “dsGFP” control from two biological replicates (N = 2). B. APL1C gene silencing abolishes APL1C signal detected by immunostaining with anti-APL1C antibody of NBM mosquito midguts. The APL1C confocal IFA signal used on fixed midguts to quantify relative abundance of APL1C is observed on the midguts from dsGFP-injected control mosquitoes (red). Nuclei and actin were stained with DAPI and Phalloidin (blue). The scale bar is 10 μm. (PDF) [file ppat.1012008.s002.pdf]

**A**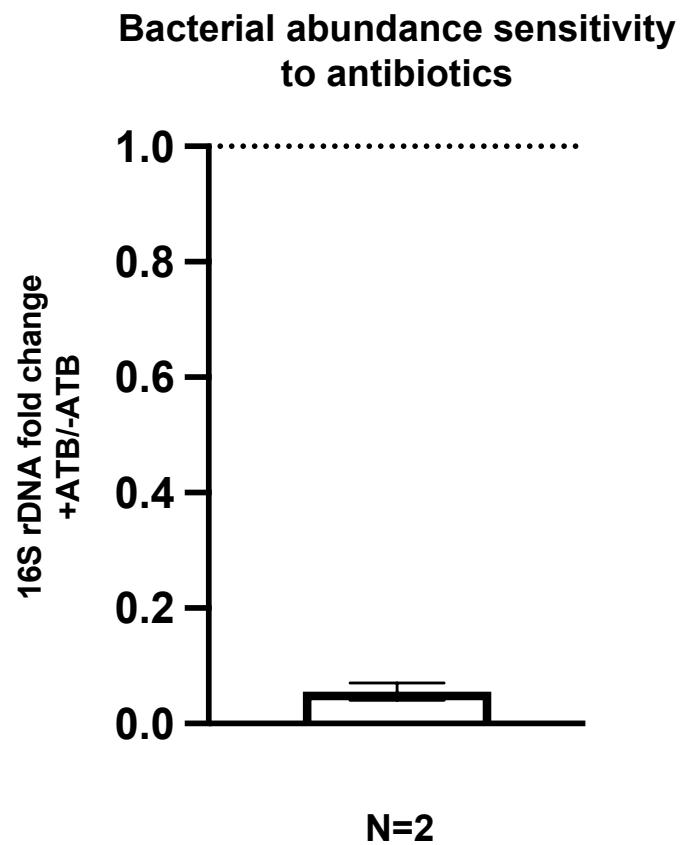**B**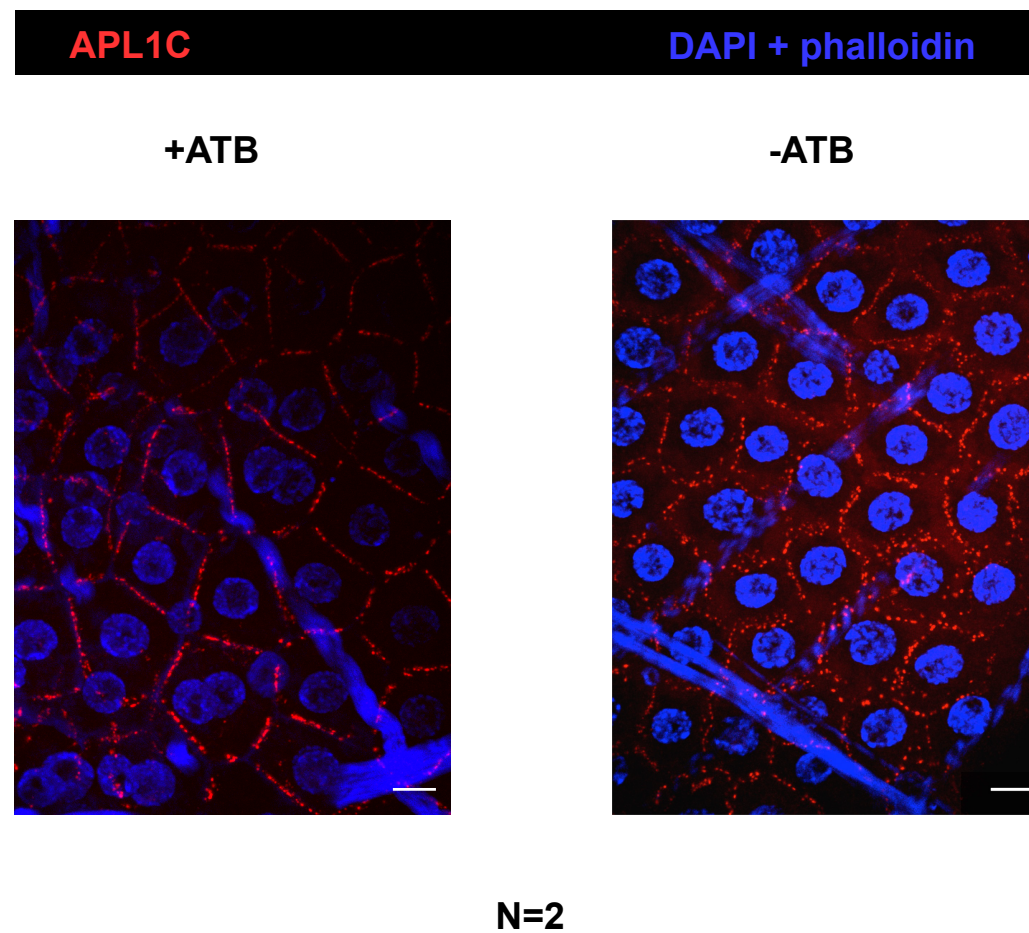

Supplement: S3 Fig — A. Bacterial abundance in mosquitoes was significantly reduced after antibiotic treatment, as confirmed by qPCR quantification of 16S ribosomal gene DNA (16S rDNA) in mosquitoes exposed (+ATB) or not (control, -ATB, depicted as a dotted line) to antibiotics at 24 h post-NBM. The ratio of normalized 16S rDNA detection in “+ATB” versus “-ATB” treatments was calculated using triplicates from the same cDNA dilution. Graph represents mean with ±SEM of the fold change between +ATB and -ATB from two biological replicates (N = 2). B. Immunostaining analysis of +ATB midguts indicates that bacterial depletion did not alter APL1C protein localization on NBM midguts. Images are representative of two independent biological replicates (N = 2, 3–7 midguts per experimental point). The scale bar is 10 μm. (PDF) [file ppat.1012008.s003.pdf]

**A**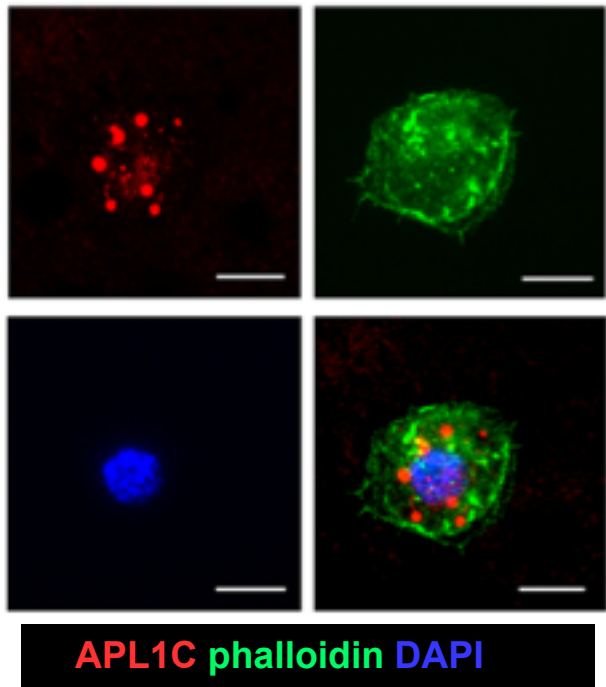**B**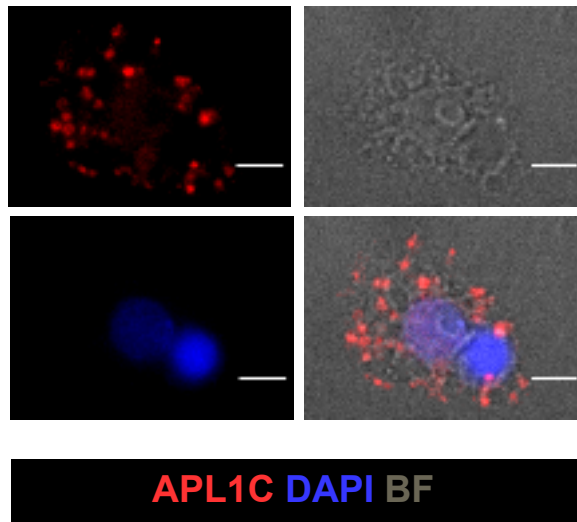**C**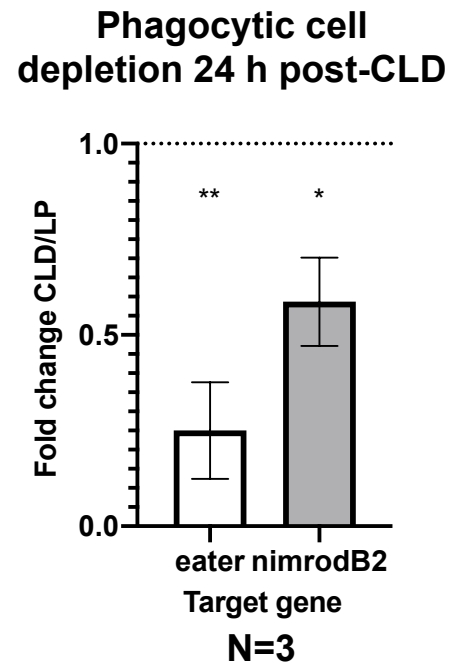

Supplement: S4 Fig — A. Immunostaining analysis of perfused hemocytes and B cultured 4a3A cells indicates that APL1C protein is localized in vesicles or vesicle-like structures (red). Cells were stained with DAPI to label nuclei (blue) and phalloidin to label actin (green). Bright field indicated as BF. The scale bar is 5 μm. C. Clodronate-mediated phagocytic cell depletion was verified by the qPCR measurement of phagocytosis markers eater and nimrodB2 between mosquitoes injected with clodronate (CLD) and control empty liposomes (LP, dotted line) at 24 h post-injection. The ratio of normalized eater or nimrodB2 cDNA detection in CLD and LP treatments was calculated using triplicates from the same cDNA dilution. Graph represents mean with ±SEM of the expression fold change between CLD and LP from three biological replicates (N = 3). Data for qPCR analysis was analyzed by unpaired t-test (significance levels of t-test p-values: * p-value<0.05; ** p-value <0.01). (PDF) [file ppat.1012008.s004.pdf]

**APL1C****GFP****DAPI + phalloidin****lu**  
**ba****lu**  
**ba**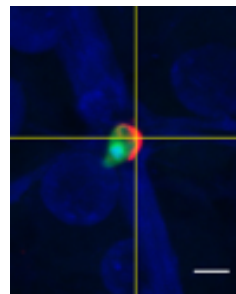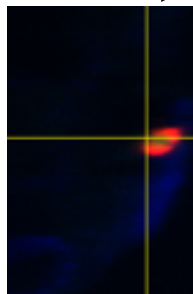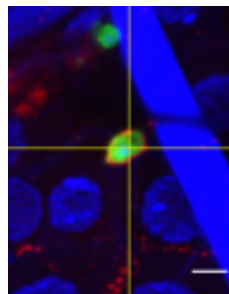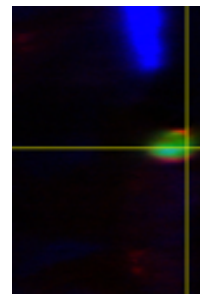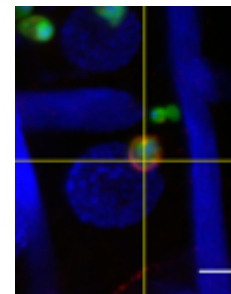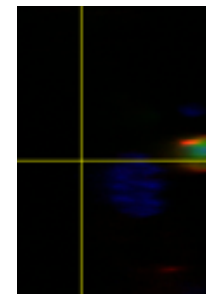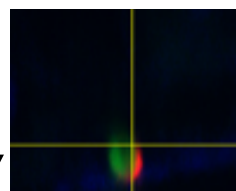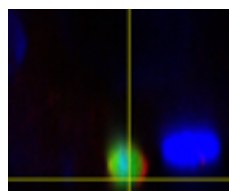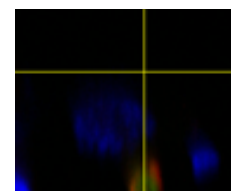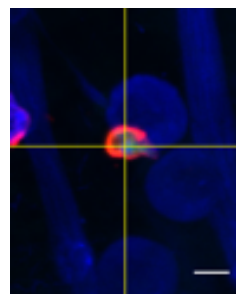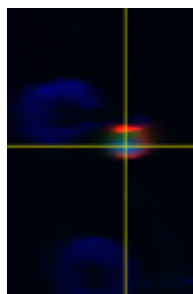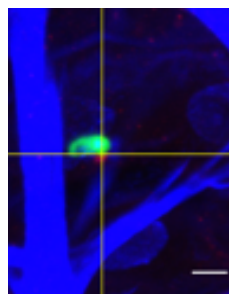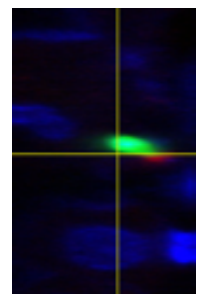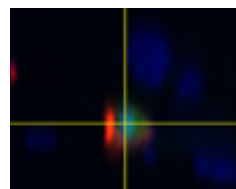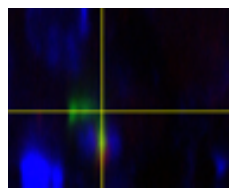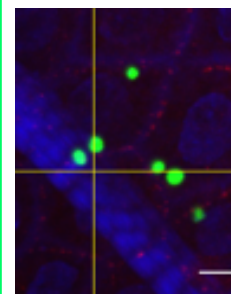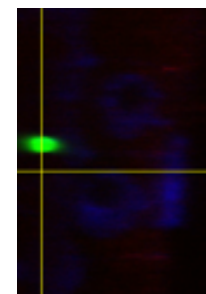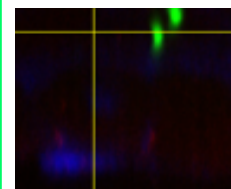

Supplement: S5 Fig — XZ and YZ orthogonal views of the confocal stack images link APL1C protein binding with parasite spatial localization in mosquito midguts. Yellow lines depict the location of the parasite, for which spatial localization is presented on the sides of the stack picture. Orientation of the midgut epithelium (lu, lumen side, ba, basolateral side) is indicated by labeled arrows on the upper left panel and applies to all panels shown. Parasites in the panels bounded by the red line were external to the basolateral side of the midgut and are labeled by APL1C protein (APL1C, red; GFP, green). Parasites in the panels bounded by the green line remained in the lumen or within epithelial cells of the mosquito midgut are not labelled with APL1C (GFP, green). The scale bar is 5 μm. (PDF) [file ppat.1012008.s005.pdf]

DAPI + phalloidin

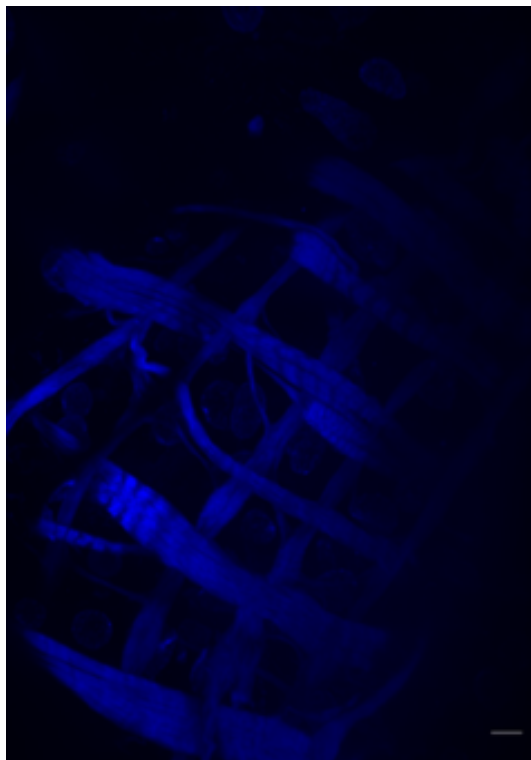

GFP

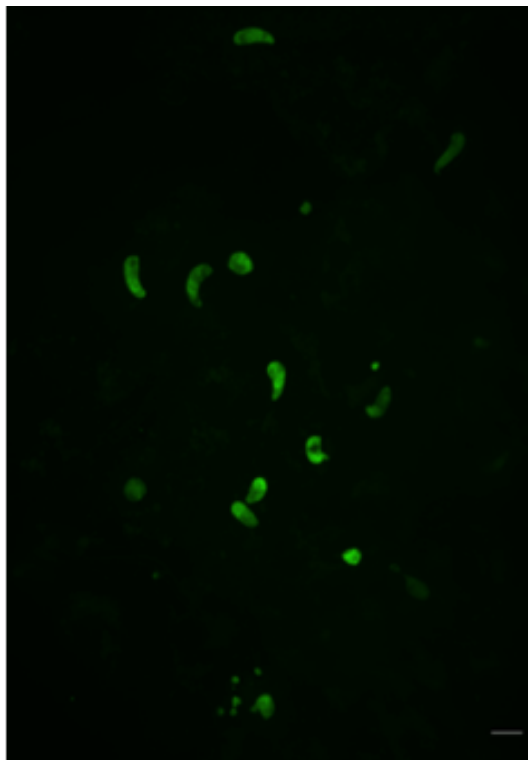

Anti-GFP

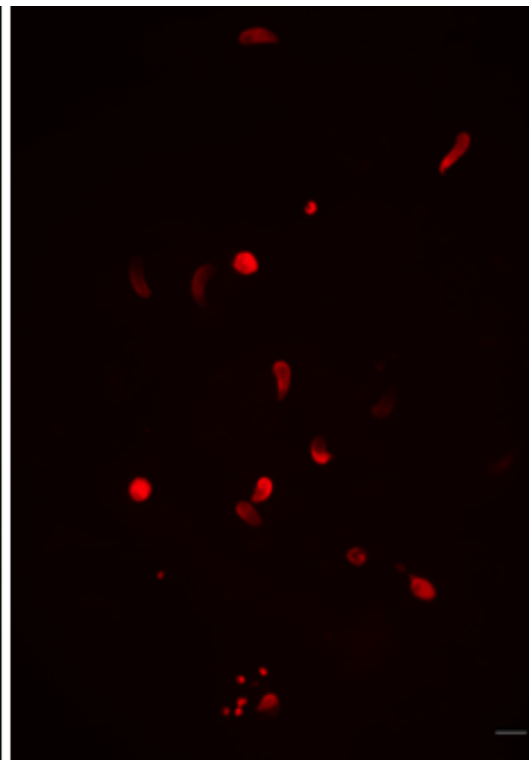

Merge

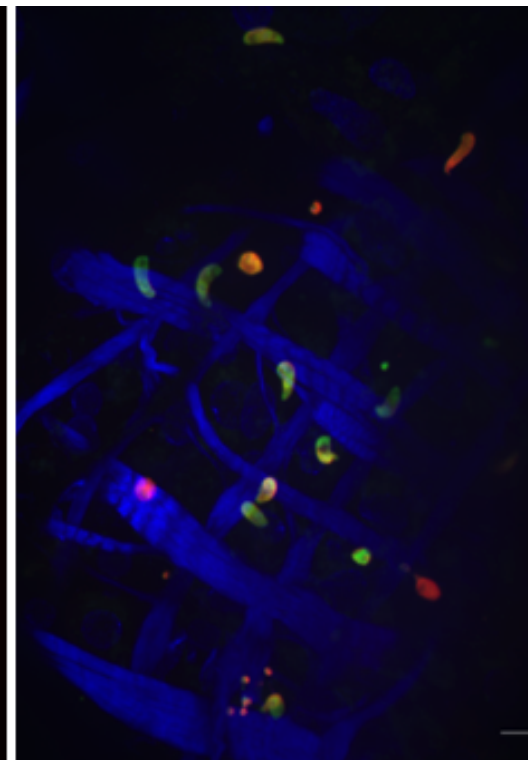

Supplement: S6 Fig — IFA of midguts collected 24 h post-IBM. Midguts were permeabilized and immunostained with anti-GFP conjugated antibody. GFP-expressing ookinetes (green) were also associated with anti-GFP antibody (red) which confirmed antibody accessibility to all parasites in mosquito midguts. Nuclei and actin were stained with DAPI and Phalloidin (blue). The scale bar is 10 μm. (PDF) [file ppat.1012008.s006.pdf]

**A**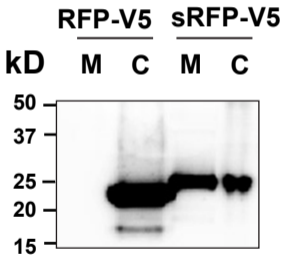**B**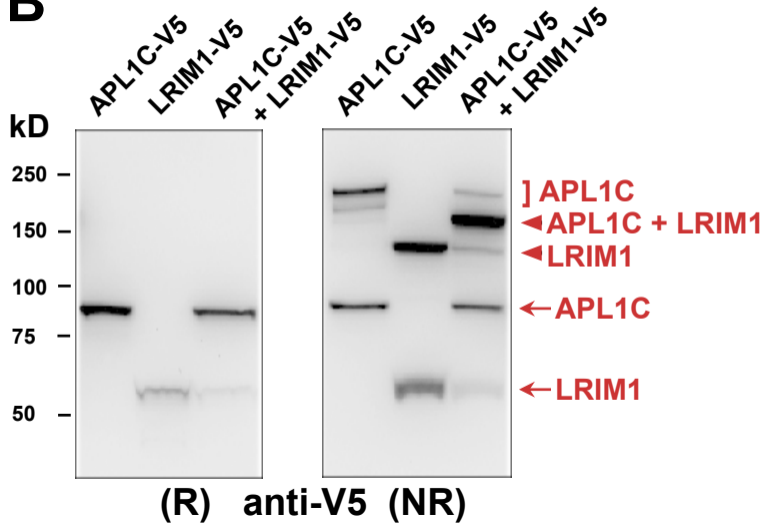

Supplement: S7 Fig — A. Immunoblot analysis of culture medium (M) and cells (C) of 4a3A cells transfected with plasmids encoding V5-tagged RFP (RFP-V5) and V5-tagged RFP fused with the signal sequence from APL1C (sRFP-V5). Immunoblot was probed with anti-V5 antibody. B. Immunoblot analysis of culture medium of 4a3A cells transfected with plasmids encoding V5-tagged APL1C and LRIM1 under reducing (R) and non-reducing (NR) conditions with anti-V5 antibody. Estimated sizes of monomeric APL1C and LRIM1 forms including V5-tag are: 88 kDa (APL1C) and 60 kDa (LRIM1), respectively. The results indicate that both APL1C-V5 and LRIM1-V5 are secreted into the culture medium. (PDF) [file ppat.1012008.s007.pdf]

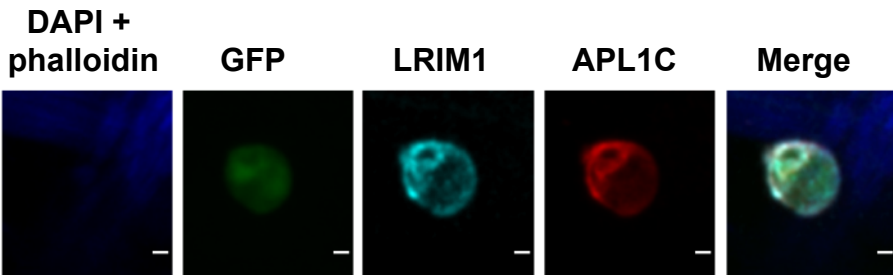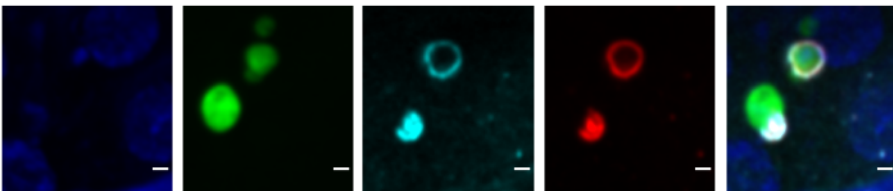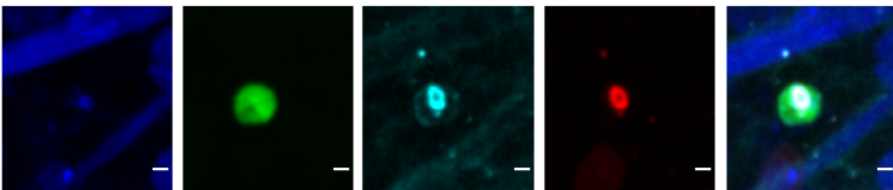

N=2

Supplement: S8 Fig — Immunostaining analysis of non-permeabilized P. berghei-infected mosquito midguts 24 h post-infection. Live, GFP-expressing (green) parasites were tested for APL1C (red) and LRIM1 (cyan) protein binding by incubation with rabbit-originated APL1C and mouse-originated LRIM1 antibodies and different fluorophore-conjugated species-specific secondary antibodies. Images shown of APL1C-positive LRIM1-positive parasites are representative of two biological replicates (N = 2). Nuclei and actin were stained with DAPI and Phalloidin (blue). The scale bar is 2 μm. (PDF) [file ppat.1012008.s008.pdf]

**A**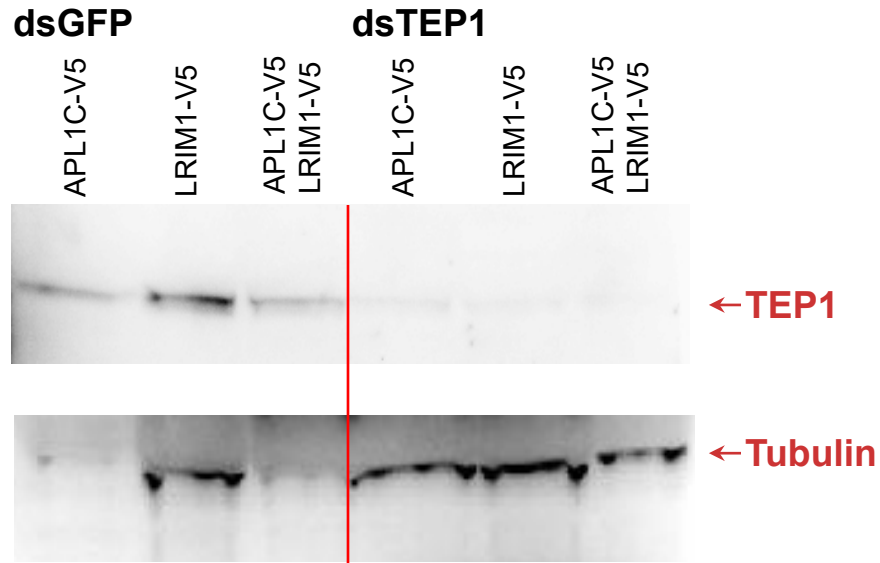**B**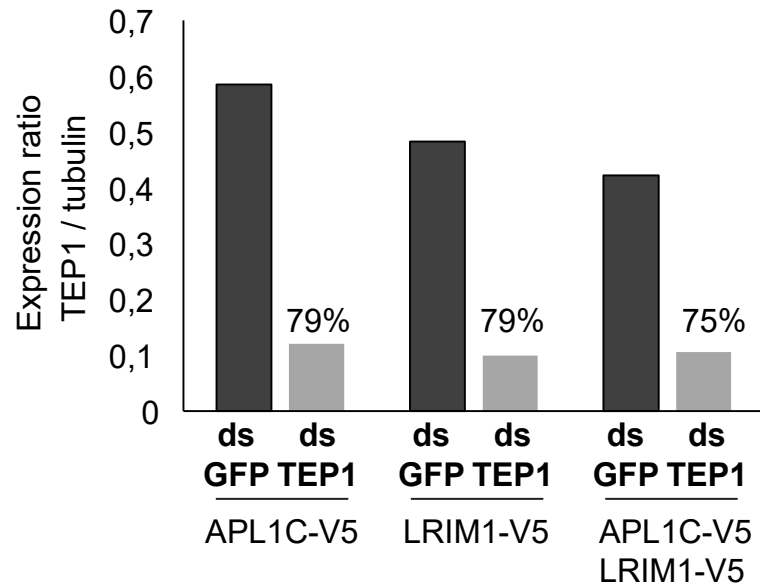

Supplement: S9 Fig — A. Western blot analysis of TEP1 protein in the 4a3A cell line. The efficiency of TEP1 gene silencing was monitored 6 d after treatment with dsTEP1 or dsGFP in cells transfected with the constructs APL1C-V5, LRIM1-V5 or both. Detection used anti-TEP1 antibody with anti-alpha tubulin antibody as a loading control. B. Quantitative analysis of TEP1 protein immunoblotting. Expression ratio of TEP1 and tubulin loading control protein levels were quantified by densitometry. For each condition, the ratio of protein levels TEP1/tubulin was calculated and the percentage of TEP1 signal reduction relative to tubulin was quantified in cells treated with dsTEP1 as compared to dsGFP. (PDF) [file ppat.1012008.s009.pdf]

A.

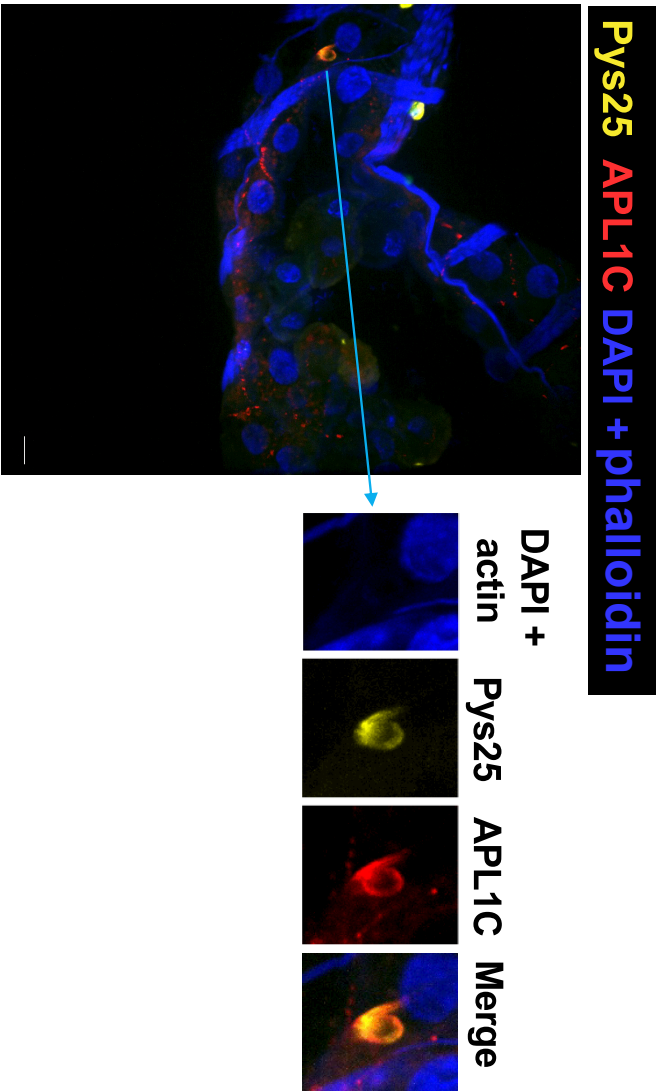

B.

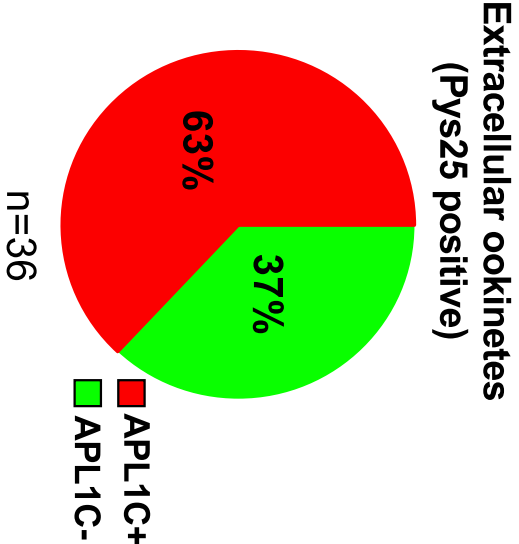

C. APL1C activity in low infection prevalence

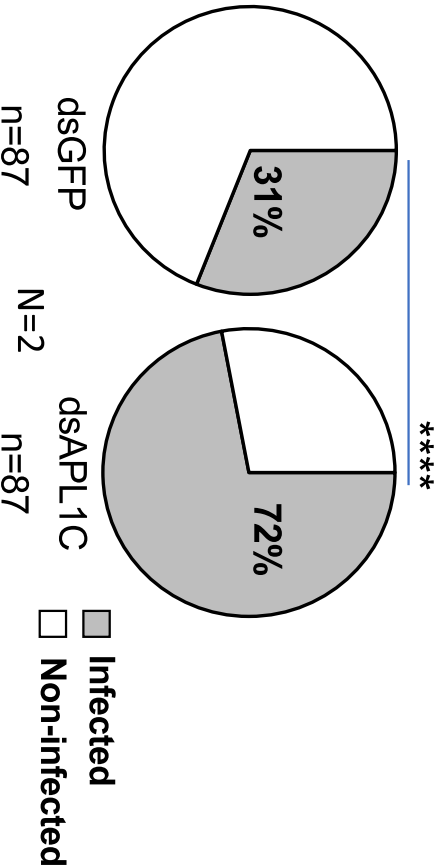

D. APL1C activity in low infection intensity

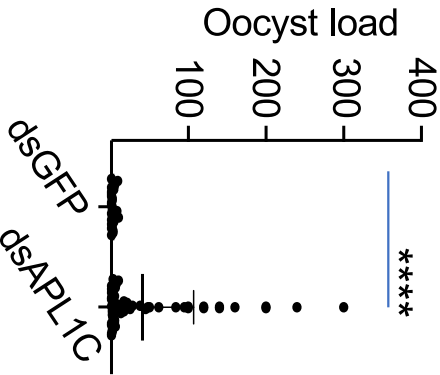

Supplement: S10 Fig — A. Immunostaining of a non-permeabilized midgut from a P. berghei low intensity infection (<10 ookinetes per midgut) 24 h post-infection detects APL1C protein binding to extracellular ookinetes. Extracellular location of parasites is indicated by staining with antibody directed against Pys25 ookinete surface protein (yellow), APL1C binding is indicated by anti-APL1C antibody staining (red). Nuclei and actin were stained with DAPI and phalloidin, respectively (blue). Scale bar, 10 μm. B. Pie chart depicts the APL1C binding outcome for all extracellular (Pys25-positive) parasites in 12 midguts with low intensity (<10 ookinetes per midgut) P. berghei infections (red slice, APL1C-positive extracellular ookinetes, green pie slice, APL1C-negative extracellular parasites). The 12 midguts infected with less than 10 ookinetes per midgut carried 36 total extracellular ookinetes of which 23 (63%) were APL1C-positive. n indicates number of live and dead ookinetes labelled with antibody against Pys25 ookinete surface protein. C. D. APL1C expression was silenced in mosquitoes infected with P. berghei at low oocyst infection prevalence (<35% infected mosquitoes in dsGFP control) and intensity (median = 2 oocysts in dsGFP control) C. Pie charts indicate that APL1C silencing causes higher infection prevalence as compared to dsGFP controls. n is the total number of dissected mosquitoes from the two replicates. D. Plot indicates that APL1C silencing causes higher infection intensity (median = 9 oocysts) as compared to dsGFP controls (median = 2 oocysts). Combined p-values from two independent replicates (N = 2) are obtained using the Fisher method; **** indicate P-value<0.0001. (PDF) [file ppat.1012008.s010.pdf]

A

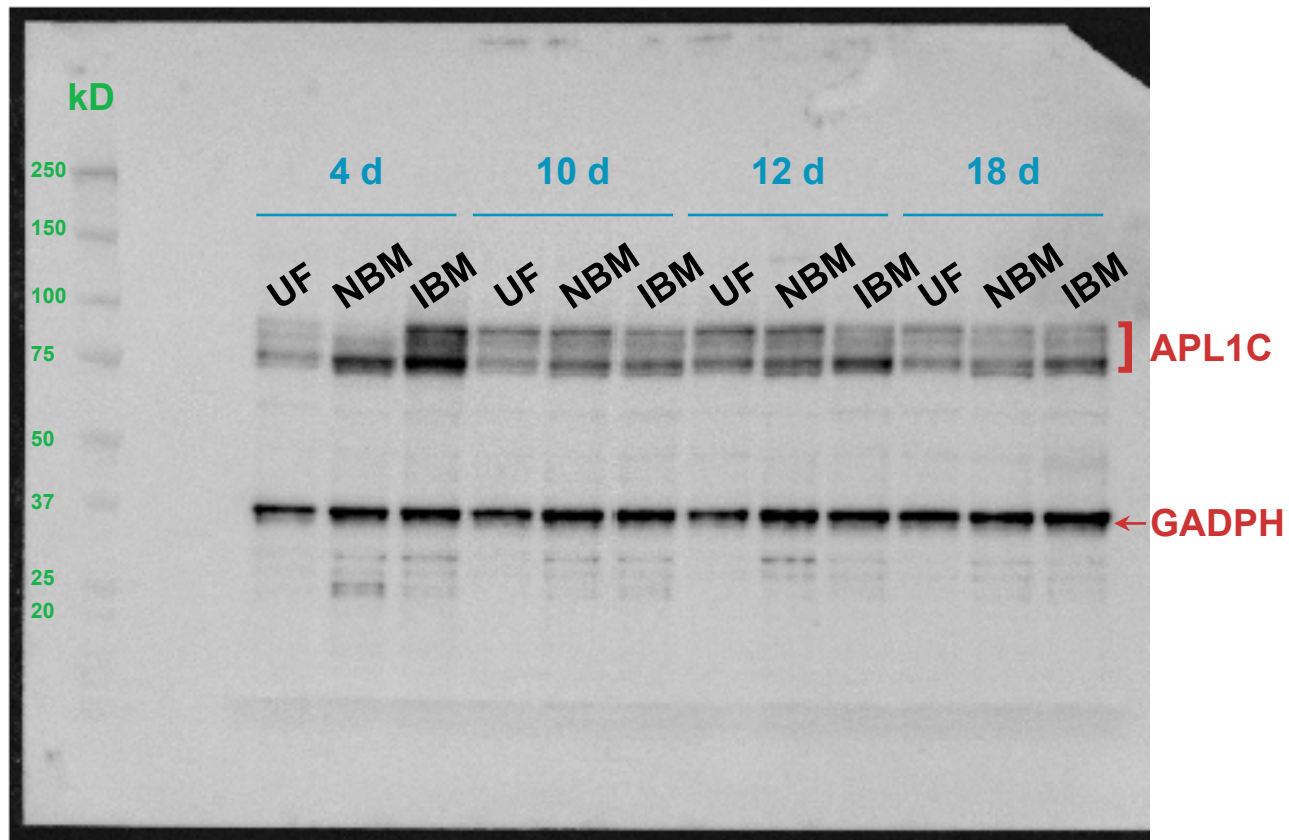

B

| Fold change   | 4 d   |       |       | 10 d  |       |       | 12 d  |       |       | 18 d  |       |       |
|---------------|-------|-------|-------|-------|-------|-------|-------|-------|-------|-------|-------|-------|
|               | Rep 1 | Rep 2 | Rep 3 | Rep 1 | Rep 2 | Rep 3 | Rep 1 | Rep 2 | Rep 3 | Rep 1 | Rep 2 | Rep 3 |
| <b>NBM/UF</b> | 188   | 182   | 126   | 86    | 202   | 117   | 76    | 66    | 104   | 124   | 103   | 95    |
| <b>IBM/UF</b> | 280   | 194   | 157   | 96    | 116   | 125   | 131   | 59    | 100   | 201   | 207   | 158   |

Supplement: S11 Fig — A. Western blot analysis of APL1C protein levels after IBM and NBM. Mosquitoes were collected at 4 d (early oocyst), 10 d (mature oocyst before sporozoites release), 12 d (early sporozoite release) and 18 d (late sporozoite release) and analyzed by western blot using anti-APL1C antibody and anti-GADPH antibody as a loading control. NBM and UF mosquitoes from corresponding time-points were used as controls. Depicted blot is representative of three independent replicates. B. Loading-control normalized APL1C protein band density ratios of IBM/UF and IBM/UF were calculated for each time point in each biological replicate. (PDF) [file ppat.1012008.s011.pdf]

**A**

APL1C transcript 10 d after  
dsAPL1C injection

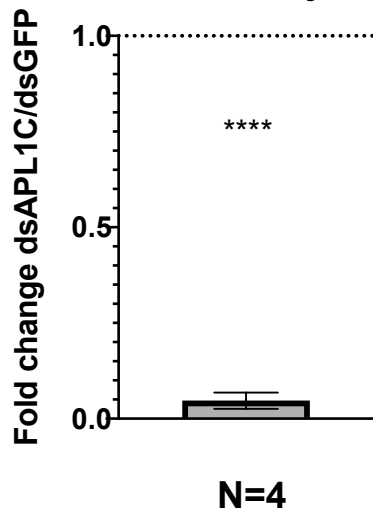**B**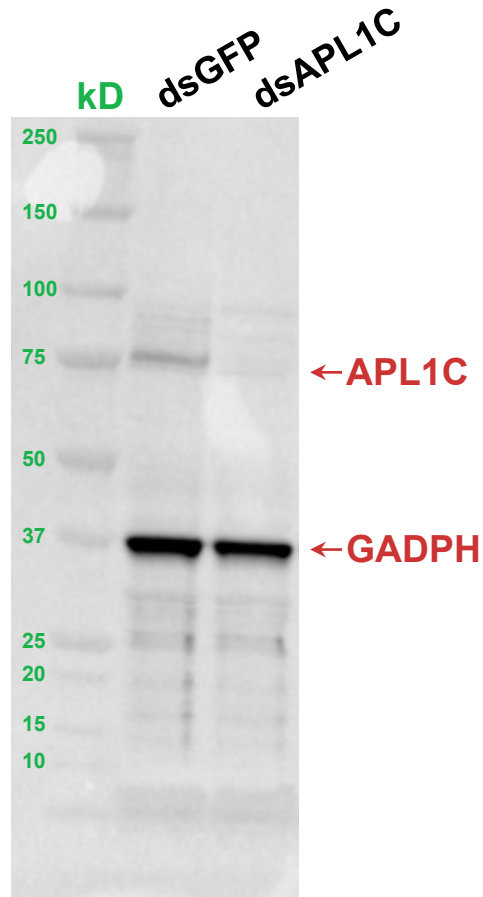**C**

APL1C protein level 10 d after  
dsAPL1C injection

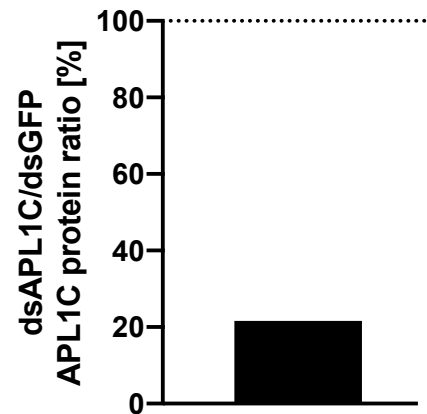

Supplement: S12 Fig — A. APL1C expression is reduced at the time of salivary gland dissections done at 10 d post dsAPL1C injection. APL1C silencing was verified by qPCR measurement between dsAPL1C and dsGFP (dotted line) treatments 10 d post-treatment. The ratio of normalized APL1C transcript in dsAPL1C versus dsGFP treatments was calculated using triplicates from the same cDNA dilution. Graph represents mean with ±SEM of the expression fold change between dsAPL1C and dsGFP control from four biological replicates (N = 4). Data for qPCR analysis was analyzed by unpaired t-test (significance level of t-test **** p-value <0.0001). B. Western blot analysis of APL1C protein level in mosquito 10 d after APL1C gene silencing. The efficiency of APL1C depletion was monitored at day 10 post dsAPL1C injection versus dsGFP control in whole mosquitoes using anti-APL1C antibody and anti-GADPH antibody as a loading control. C. APL1C protein level is still decreased 10 d after dsAPL1C injection. The loading control normalized APL1C protein band densities were compared between dsAPL1C and dsGFP (dotted line) injected mosquitoes. (PDF) [file ppat.1012008.s012.pdf]

**A****dsGFP**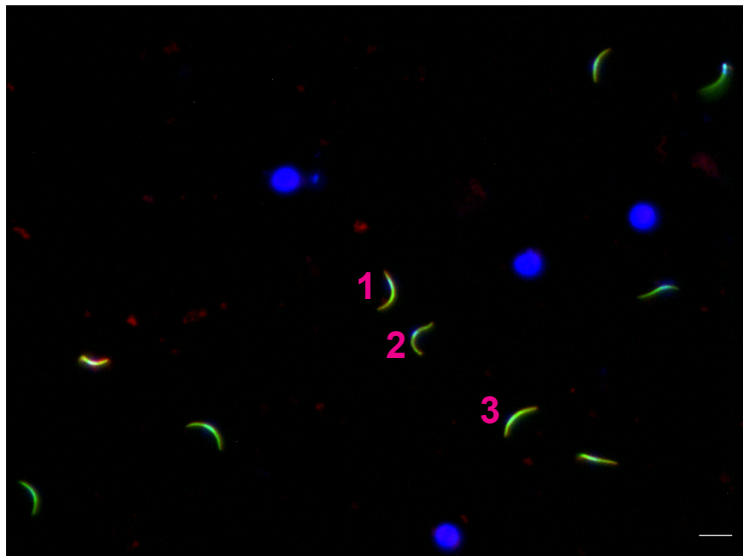**DAPI****GFP****APL1C****Merge****1**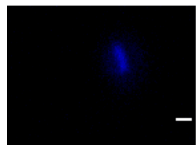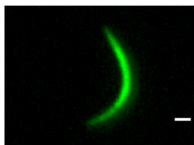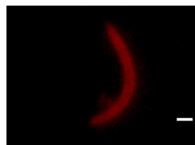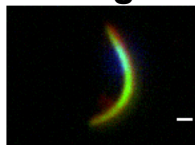**2**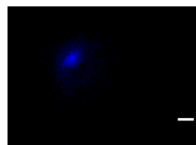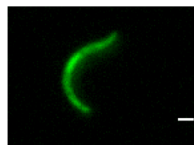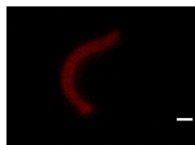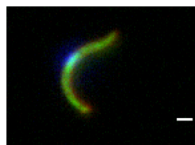**3**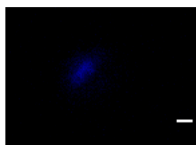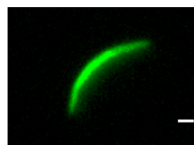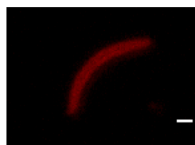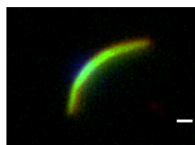**B****dsAPL1C**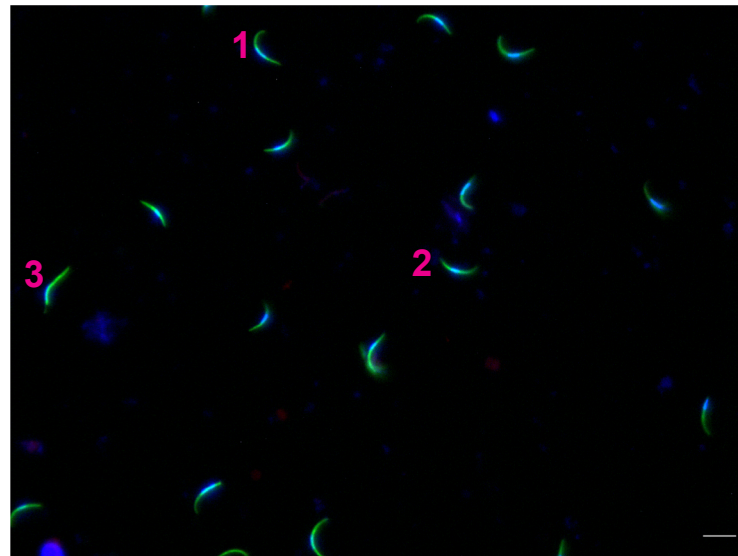**DAPI****GFP****APL1C****Merge****1**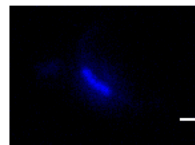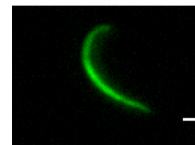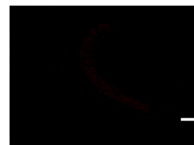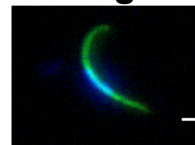**2**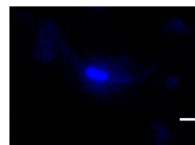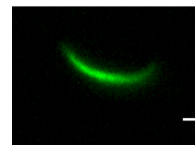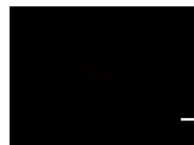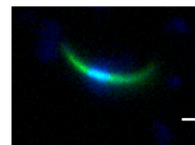**3**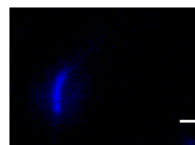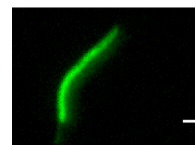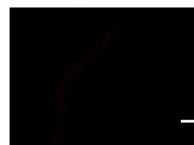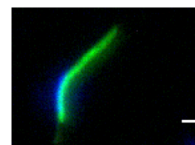

Supplement: S14 Fig — A. Sporozoites perfused from control dsGFP-treated mosquitoes are labelled by APL1C protein. The top panel depicts merged images of representative sporozoites (scale bar 10 μm), whereas the images below show 3 channels of enlarged projections of sporozoites (scale bar 2 μm), indicated by the numbers on the merged image. B. Sporozoites perfused from dsAPL1C-treated mosquitoes are not labelled by APL1C protein. The top panel depicts merged images of representative sporozoites (scale bar 10 μm), whereas the images below show 3 channels of enlarged projections of sporozoites (scale bar 2 μm), indicated by the numbers on the merge picture. (PDF) [file ppat.1012008.s014.pdf]
