# Supplementary material for: The Anopheles leucine-rich repeat protein APL1C is a pathogen binding factor recognizing Plasmodium ookinetes and sporozoites
Source: PLoS Pathog. 2024 Feb 14;20(2):e1012008. doi: 10.1371/journal.ppat.1012008 (PMC10898737; doi:10.1371/journal.ppat.1012008)

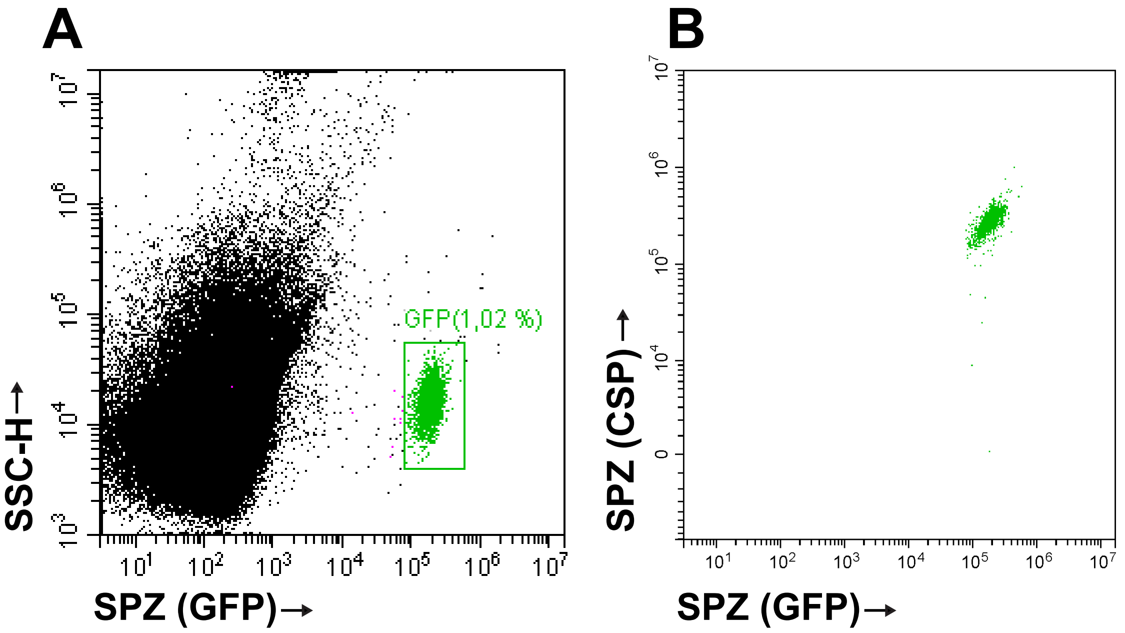

E

SPZ-APL1C labelling	12 d			15 d			22 d	
	Rep 1	Rep 2	Rep 3	Rep 1	Rep 2	Rep 3	Rep 1	Rep 2
APL1C+ [%]	80	84	88	76	71	35	68	73
APL1C+ [SPZ nr]	37	343	43	1792	1790	43	203	1130
APL1C- [%]	20	16	12	24	29	65	32	27
APL1C- [SPZ nr]	9	67	6	564	716	81	95	417

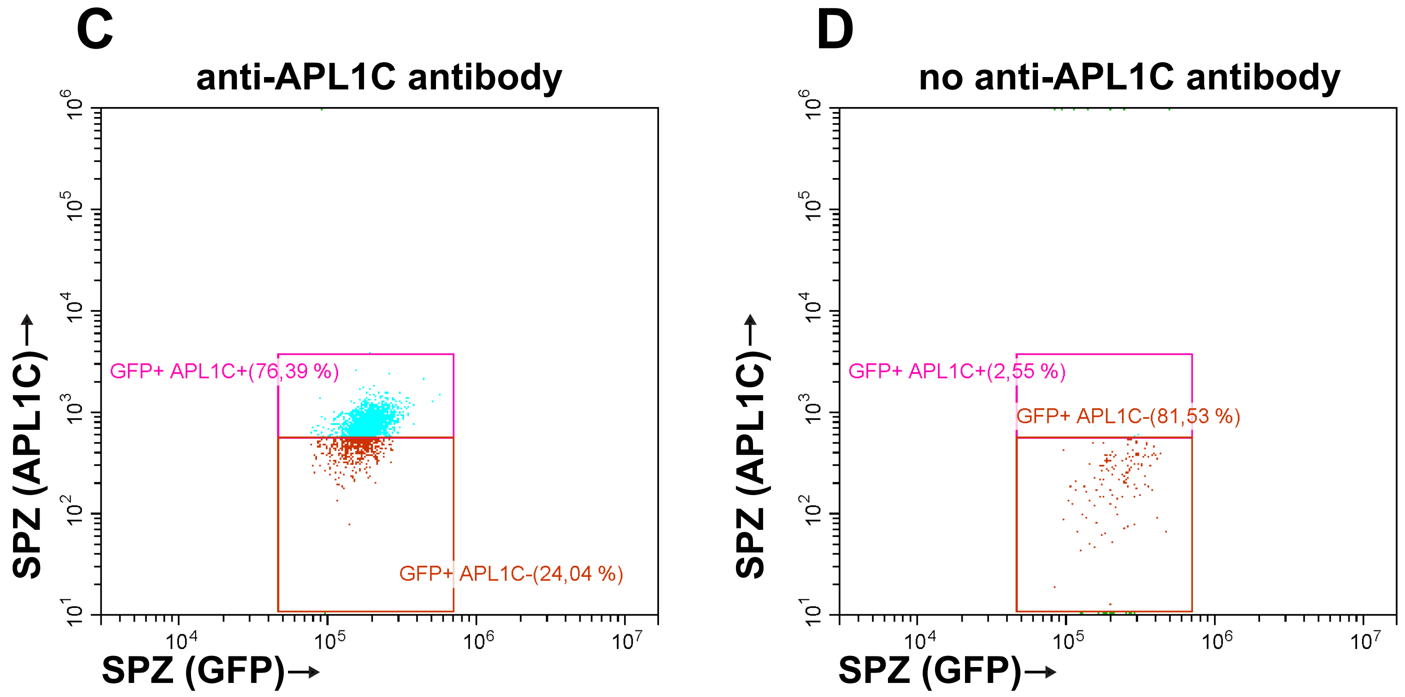

Supplement: S13 Fig — A. GFP-expressing sporozoites perfused in mosquito hemolymph were gated in a side scatter height (SSC-H) versus GFP in a dot plot graph. B. The sporozoite population that expressed GFP was confirmed by CSP staining, which yielded the same number of events. C. APL1C gating strategy. The fluorescence corresponding to APL1C staining was determined from the GFP population. D. APL1C gating control to determine APL1C signal threshold. Sporozoites were incubated with an antibody different from anti-APL1C (mouse anti-CSP) and Alexa Fluor 555-conjugated anti-rabbit secondary antibody. E. APL1C labelling of hemolymph perfused sporozoites, at 12 d, 15 d and 22 d post mosquito infection. Sporozoites in each replicate are reported as percentage [%] or number of APL1C labelled sporozoites out of global sporozoites population per each collection timepoint. (PDF) [file ppat.1012008.s013.pdf]
