# Supplementary material for: The Anopheles leucine-rich repeat protein APL1C is a pathogen binding factor recognizing Plasmodium ookinetes and sporozoites
Source: PLoS Pathog. 2024 Feb 14;20(2):e1012008. doi: 10.1371/journal.ppat.1012008 (PMC10898737; doi:10.1371/journal.ppat.1012008)
Supplement: S15 Fig — A. APL1C was silenced by dsRNA treatment of mosquitoes at 9 d post-IBM, prior to sporozoite release from oocysts. At 17 d post-IBM, during sporozoite release, mosquitoes were perfused individually and the number of circulating sporozoites per individual mosquito was counted by flow cytometry in mosquitoes treated with dsAPL1C or control dsGFP. Graph presents the sporozoite number in perfused individuals between dsAPL1C- and dsGFP-treated mosquitoes. Each point represents a single perfused individual, bars represent mean with ±SEM. Sample sizes (N) show the number of independent replicate experiments, (n) the total number of perfused individuals across replicates. Data were compared between the two conditions by Mann-Whitney test. All statistical differences tested independently within replicates (individual p-values in S1 Table) (significance level of Mann-Whitney n.s. = not significant). B. APL1C silencing by dsAPL1C treatment 9 d post-IBM was still efficient at the time of mosquito perfusion, 17 d post-IBM, as verified by qPCR. The ratio of the normalized APL1C cDNA detection in dsAPL1C versus dsGFP treatments was calculated using triplicates from the same cDNA dilution. Graph represents mean with ±SEM of the transcript abundance fold change between dsAPL1C and dsGFP samples from independent biological replicates (N). Data for qPCR analysis was analyzed by unpaired t-test (significance levels of t-test p-values: *** p-value <0.001). C. Test of LRIM1 function for hemocoel sporozoite numbers. Description as in panel A but substituting dsLRIM1 in place of dsAPL1C. D. LRIM1 silencing by dsLRIM1 treatment 9 d post-IBM was still efficient at the time of mosquito perfusion, 17 d post-IBM, as verified by qPCR. Description as in panel B but substituting dsLRIM1 in place of dsAPL1C. (PDF) [file ppat.1012008.s015.pdf]

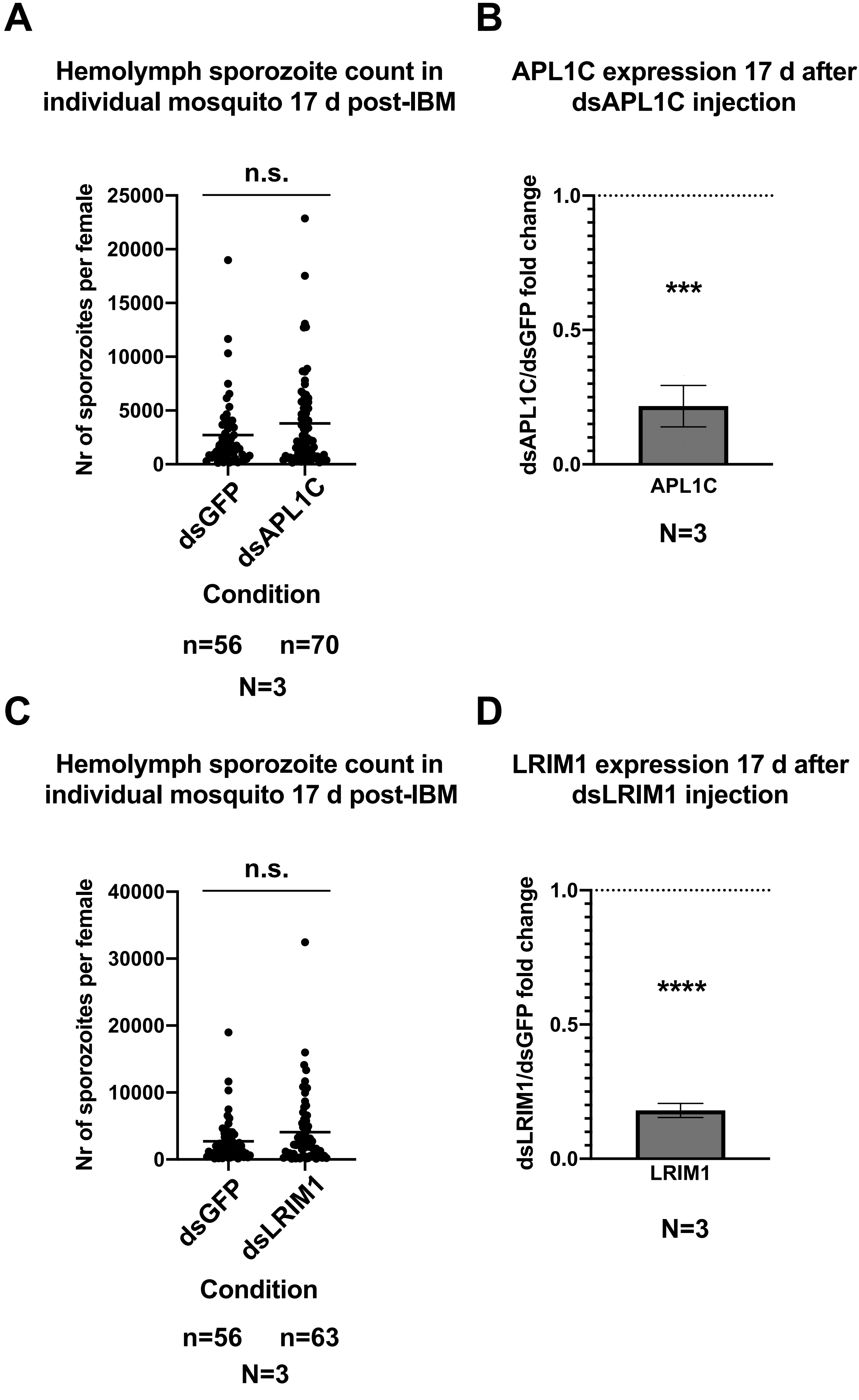

A
Hemolymph sporozoite count in individual mosquito 17 d post-IBM
n.s.
Nr of sporozoites per female
25000
20000
15000
10000
5000
0
dsGFP
dsAPL1C
Condition
n=56 n=70
N=3
B
APL1C expression 17 d after dsAPL1C injection
dsAPL1C/dsGFP fold change
1.0
0.5
0.0
***
APL1C
N=3
C
Hemolymph sporozoite count in individual mosquito 17 d post-IBM
n.s.
Nr of sporozoites per female
40000
30000
20000
10000
0
dsGFP
dsLRIM1
Condition
n=56 n=63
N=3
D
LRIM1 expression 17 d after dsLRIM1 injection
dsLRIM1/dsGFP fold change
1.0
0.5
0.0
****
LRIM1
N=3
