# Supplementary material for: The Anopheles leucine-rich repeat protein APL1C is a pathogen binding factor recognizing Plasmodium ookinetes and sporozoites
Source: PLoS Pathog. 2024 Feb 14;20(2):e1012008. doi: 10.1371/journal.ppat.1012008 (PMC10898737; doi:10.1371/journal.ppat.1012008)
Supplement: S16 Fig — A. TEP1 and TEP3 expression is efficiently silenced at the time of salivary gland dissection (10 d post-dsTEP1, dsTEP3 or dsTEP1/TEP3 injections). TEP1 and TEP3 silencing was verified by the qPCR measurement. The ratio of the normalized TEP1 or TEP3 cDNA detection in dsTEP1, dsTEP3 or dsTEP1/TEP3 versus dsGFP treatments was calculated using triplicates from the same cDNA dilution. Graphs represent mean with ±SEM of the expression fold change from independent biological replicates (N). Data for qPCR analysis was analyzed by unpaired t-test (significance levels of t-test p-values: ** p-value <0.01; *** p-value <0.001). B. TEP1, TEP3 or simultaneous TEP1/TEP3 depletion does not influence sporozoite salivary gland infection. The percentage of sporozoite-infected salivary glands (gray) or non-infected (white) in dsTEP1, dsTEP3 or dsTEP1/TEP3 mosquitoes and dsGFP injected control are shown in pie charts as the mean percentage obtained from independent number of biological replicates (N). Prevalence from each replicate was compared between the two conditions by chi-square test. All statistical differences were first tested independently within replicates (individual p-values in S2 Table), and if individual replicates showed a common trend of change, individual p-values were combined using the meta-analytical approach of Fisher (significance level of chi-square n.s. = not significant). (PDF) [file ppat.1012008.s016.pdf]

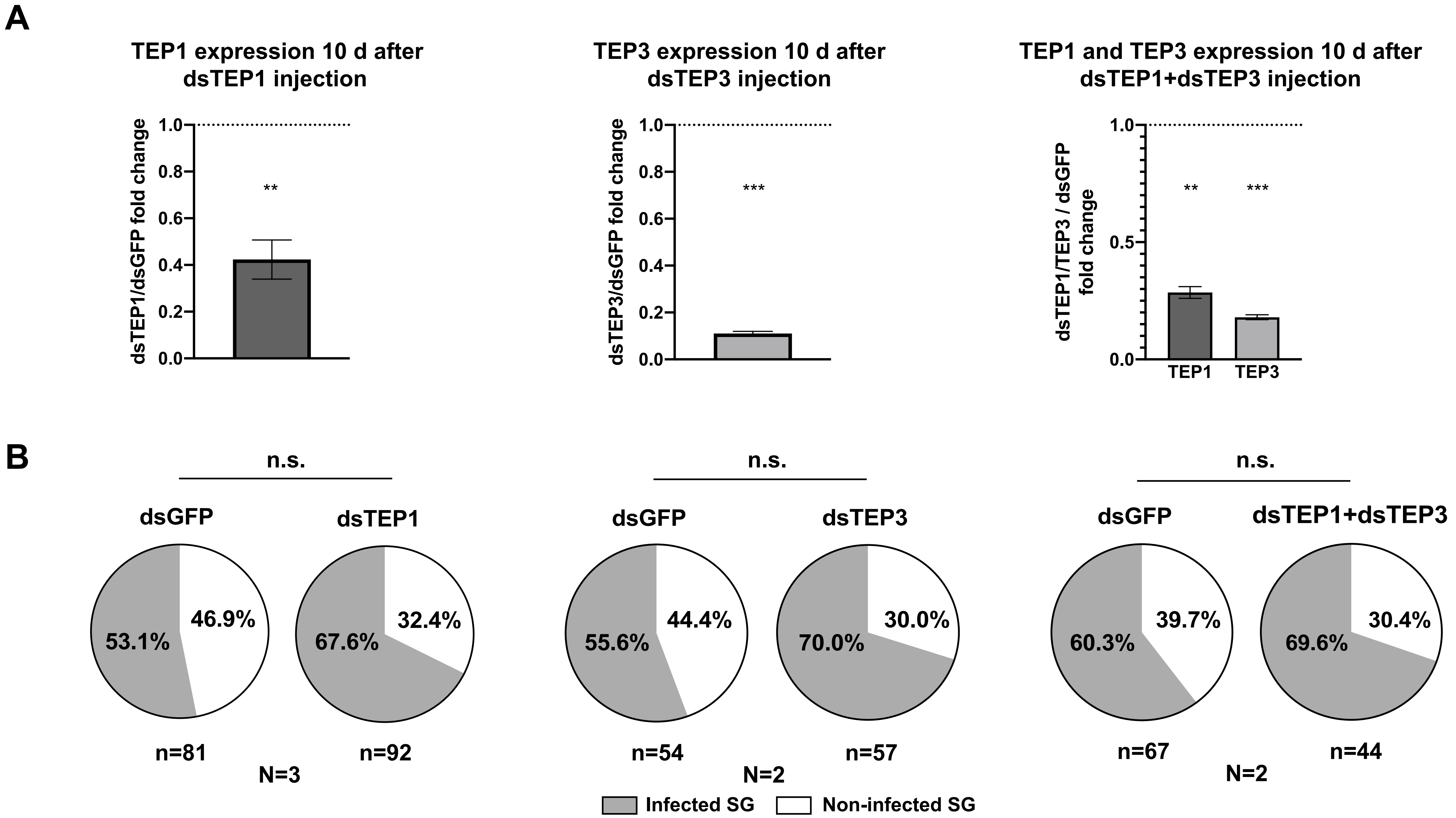
A
TEP1 expression 10 d after dsTEP1 injection
dsTEP1/dsGFP fold change
1.0
0.8
0.6
0.4
0.2
0.0
**
TEP3 expression 10 d after dsTEP3 injection
dsTEP3/dsGFP fold change
***
TEP1 and TEP3 expression 10 d after dsTEP1+dsTEP3 injection
dsTEP1/TEP3 / dsGFP fold change
0.5
**
***
TEP1
TEP3
B
n.s.
dsGFP
dsTEP1
53.1%
46.9%
67.6%
32.4%
n=81
n=92
N=3
dsTEP3
55.6%
44.4%
70.0%
30.0%
n=54
n=57
N=2
dsTEP1+dsTEP3
60.3%
39.7%
69.6%
30.4%
n=67
n=44
Infected SG
Non-infected SG
